# Implementation of a hospital deprescribing behaviour change intervention, the CompreHensive geriAtRician-led MEdication Review (CHARMER) trial: a process evaluation protocol

**DOI:** 10.1136/bmjopen-2025-111152

**Published:** 2026-06-02

**Authors:** Jacqueline M Martin-Kerry, Debi Bhattacharya, Johanna Taylor, Ian Kellar, Bethany Atkins, Charlotte E L Jones, Elizabeth M Bywater-Florance, Victoria L Keevil, Allan B Clark, David John Wright, David Phillip Alldred, Sion Scott

**Affiliations:** 1 School of Health Sciences, University of East Anglia, Norwich, UK; 2 Department of Health Sciences, University of York, York, UK; 3 School of Psychology, The University of Sheffield, Sheffield, UK; 4 Department of Clinical and Biomedical Sciences, University of Exeter, Exeter, UK; 5 Healthcare for Older People, Royal Devon University Healthcare NHS Foundation Trust, Exeter, England, UK; 6 Norwich Clinical Trials Unit, Norwich Medical School, University of East Anglia, Norwich, UK; 7 School of Healthcare, University of Leicester, Leicester, UK; 8 School of Healthcare, University of Leeds, Leeds, UK; 9 Pharmacy Department, Norfolk and Norwich University Hospitals NHS Foundation Trust, Norwich, UK

**Keywords:** Polypharmacy, GERIATRIC MEDICINE, Behavior, Hospitals, Physicians, Pharmacists

## Abstract

**Introduction:**

Proactive deprescribing is the process of stopping a medicine and comprises four steps: (1) identify a patient for potential stop of a medicine, (2) evaluate a patient for potential stop of a medicine, (3) stop a medicine and (4) monitor after stopping.

The CHARMER (CompreHensive geriAtRician-led MEdication Review) trial is a stepped-wedge design to evaluate the effectiveness and cost-effectiveness of a behaviour change intervention to increase proactive deprescribing in hospitals. The CHARMER intervention comprises a deprescribing action plan, deprescribing briefings, videos of successful deprescribing consultations, deprescribing case studies workshop and a deprescribing performance dashboard. The process evaluation will explore trial processes, CHARMER intervention implementation, CHARMER behavioural mechanisms of action and contextual factors influencing these aspects.

**Methods and analysis:**

The convergent parallel design process evaluation will follow the UK Medical Research Council guidance. We will interview: staff involved in CHARMER implementation, geriatricians and pharmacists who receive the intervention and research delivery staff involved in patient/carer recruitment and data collection. We will also interview patients/carers and primary care practitioners. Interviews will be supplemented with recordings of implementation activities and completed implementation manuals. Questionnaires will capture the extent to which the four proactive deprescribing steps are enacted by intervention recipients, measure the behavioural mechanisms by which the CHARMER intervention operates and capture the patient experience of proactive deprescribing. Qualitative data will be analysed thematically and then mapped to Normalisation Process Theory to explore implementation and the Theoretical Domains Framework to explore behaviour change. Most quantitative data will be analysed descriptively; however, changes in staff questionnaire responses preintervention and postintervention will be analysed using a Mann-Whitney test. We will triangulate qualitative and quantitative findings to explain intervention effects.

**Ethics and dissemination:**

Ethical and governance approvals have been obtained by the Wales 2 Research Ethics Committee and the Health Research Authority, respectively. The dissemination strategy will be underpinned by the evidence-based Guide to Disseminating Research (GuiDiR) targeting healthcare practitioners, policy makers and patient-facing organisations.

**Trial registration number:**

ISRCTN13248281.

**STRENGTHS AND LIMITATIONS OF THIS STUDY**

- The process evaluation builds on learning from a comprehensive process evaluation in the CHARMER (CompreHensive geriAtRician-led MEdication Review) feasibility study.
- The study incorporates two theoretical frameworks to explore CHARMER intervention implementation (Normalisation Process Theory) and behavioural mechanisms of action (Theoretical Domains Framework).
- Not able to observe all intervention activities (briefings and deprescribing performance dashboard) due to patient confidentiality but will explore how these are experienced in interviews.
- The fidelity framework will be operationalised to ensure we can systematically undertake fidelity for each CHARMER intervention component.
- Extensive work with patient and public involvement team members has informed several trial aspects, including how we engage with older people and their carers to seek their experiences of having a medicine stopped in hospital.

## Introduction

 Over 50% of older people admitted to hospital are taking a prescribed medicine where the risk of harm outweighs the chances of benefit^1^ predisposing them to adverse outcomes including morbidity, rehospitalisation and mortality.^1^ Proactive deprescribing is the process of stopping a medicine before it causes harm and is core to reducing medicine-related harm.^2^ Despite being a component of good prescribing practice, only 1% of older people in hospital have a medicine proactively deprescribed during their admission^3^ representing a significant missed opportunity.

The CompreHensive geriAtRician-led MEdication Review (CHARMER) intervention is a five-component deprescribing intervention designed to address the barriers and enablers to hospital proactive deprescribing identified in a focus group study with 54 geriatricians and pharmacists from four hospitals in England.^4^ The CHARMER intervention is underpinned by behavioural science and was co-designed with geriatricians, pharmacists, patients, carers and hospital staff involved in implementing new services.^5^ It comprises a deprescribing action plan, deprescribing briefings, a workshop demonstrating successful deprescribing consultations, a deprescribing case studies workshop and a national deprescribing performance dashboard.

We undertook the CHARMER feasibility study across four hospitals in England in 2022.^6 7^ A process evaluation embedded within the feasibility study aimed to inform refinement of the intervention, its implementation and trial processes to inform the design of a definitive trial.^8^ The quality and completeness of trial data within the CHARMER feasibility study were acceptable and the process evaluation found that the intervention was acceptable. Hospitals experienced challenges with implementing CHARMER because research infrastructure and processes in National Health Service (NHS) hospitals are configured to support Clinical Trials of an Investigational Medicinal Product, rather than trials such as CHARMER that evaluate complex behaviour change interventions targeting practitioner behaviour. The challenges faced by hospitals when implementing the CHARMER intervention within the feasibility study are reported separately.^7^ The main challenges were that sites needed more than the allocated 4 weeks to implement CHARMER, delivering a hospital-level action plan was not achievable, benchmarking reports were not always viewed by geriatricians and pharmacists, delivering the workshops was challenging and some geriatricians did not engage with the workshop videos. We aimed to address these challenges with modifications to the implementation period and funding a staff member to lead the implementation at each site, as well as refining guidance about how to implement each of the five CHARMER intervention components.

The Health Innovation Network is commissioned by the UK Government’s Department of Health and Social Care to support the NHS in adopting new innovations.^9^ We have commissioned Health Innovation East to facilitate implementation of the CHARMER intervention in hospitals in the definitive trial, thus mimicking the NHS’s usual process for innovation adoption and addressing the challenges identified in the feasibility study. This partnership between a trial team and Health Innovation Network is novel and may provide a model for future trials of complex interventions.

The CHARMER definitive trial is a stepped-wedge cluster randomised trial which aims to evaluate the effectiveness and cost-effectiveness of the CHARMER intervention at reducing 90-day hospital readmissions, the trial’s primary outcome measure (ISRCTN13238281).

Process evaluations embedded within a definitive trial aim to explain outcomes and variation in outcomes. This is achieved by understanding how an intervention was implemented, how it produced its effects and which factors moderate and mediate these effects. These data inform subsequent ‘real world’ adoption and implementation of interventions that are deemed to be effective and cost-effective. The use of theory to understand intervention effects within process evaluations is widely recognised.

This protocol describes the methods of a process evaluation embedded within a stepped-wedge randomised trial to evaluate the effectiveness and cost-effectiveness of the CHARMER intervention. The process evaluation aims to describe the implementation, receipt and impact of the CHARMER intervention.

## Methods

### CHARMER definitive trial design

**Figure 1:**
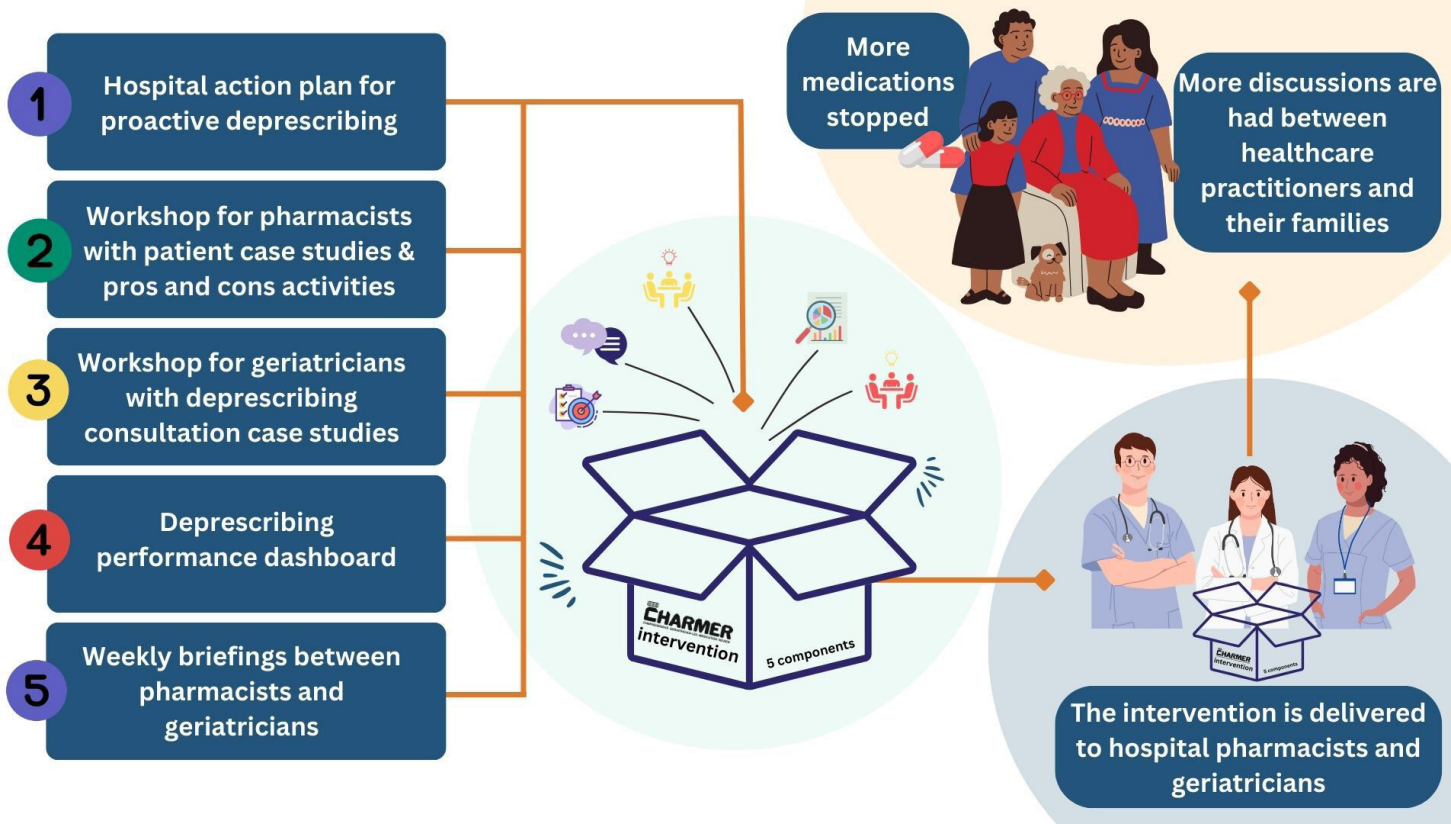
Overview of CHARMER intervention components and its desired impact. CHARMER, CompreHensive geriAtRician-led MEdication Review.

The design of the CHARMER definitive trial is comprehensively reported elsewhere (manuscript submitted and under review) and a summary is provided in Figure 1. The trial began in February 2024 and will conclude on 31 October 2025. The process evaluation began in February 2024, final data collection will be complete in January 2026 and the analysis will be completed in August 2026.

CHARMER is a stepped-wedge randomised trial across 24 NHS hospitals in England over 18 months. 20 hospitals will implement the CHARMER intervention on Older People’s Medicine (OPM) wards and collect data; four reserve hospitals will collect data only, unless they are required to become an intervention hospital prompted by the withdrawal of an existing intervention hospital. A principal investigator (PI), who is either a geriatrician or pharmacist, will assume overall responsibility for trial conduct at each hospital, including intervention implementation and data collection.

The CHARMER intervention will be delivered to approximately 200 geriatricians and pharmacists and data to evaluate its effects will be collected for approximately 44 000 patients admitted to study wards. The trial is powered to detect a 3% reduction in its primary outcome measure of readmission to hospital within 90days of discharge.

We will obtain anonymised routinely collected data, including readmissions for all patients admitted during the study period. Additionally, we will recruit patients and carers during two enhanced data collection periods, each of 1 month, during control and intervention. These will provide data for secondary outcomes and the process evaluation.

### CHARMER intervention, programme theory and logic model

**Figure 2:**
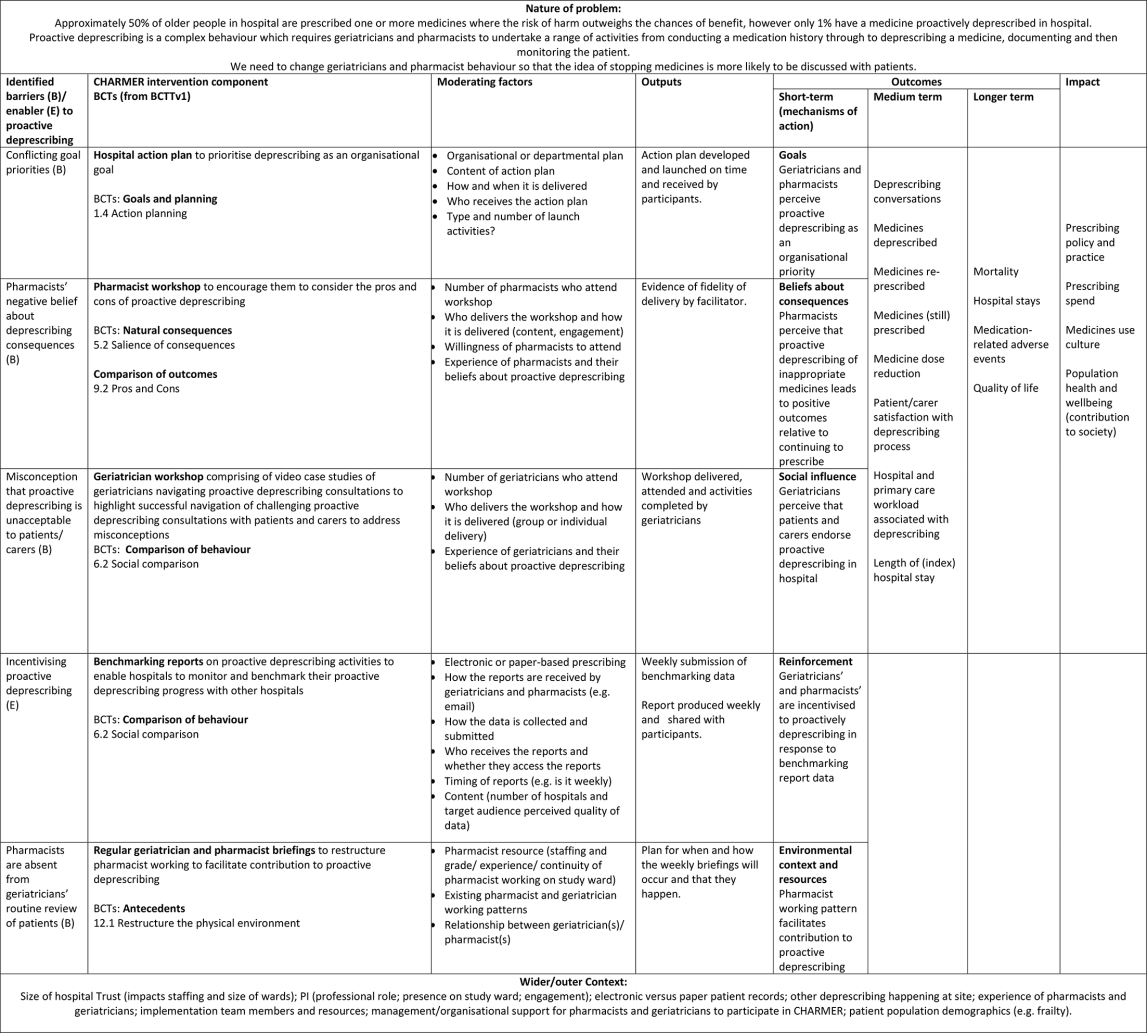
CHARMER logic model. CHARMER, CompreHensive geriAtRician-led MEdication Review; BCT, Behaviour Change Technique; NHS, National Health Service; NIHR, National Institute for Health and Care Research.

The development and content of the CHARMER intervention are comprehensively reported elsewhere.^5^ Figure 1 provides a summary of the CHARMER intervention and figure 2 provides a logic model. The logic model was refined based on learning from the CHARMER feasibility study process evaluation (manuscript under review) and hypothesises the mechanisms of action by which the intervention may address geriatricians’ and pharmacists’ barriers and enablers to proactive deprescribing. We will further refine the logic model based on learning from the definitive trial process evaluation.

### CHARMER intervention implementation

Hospitals will have 12 weeks to implement the CHARMER intervention. We will provide funding for a project manager 1 day per week for 14 weeks to lead implementation under the PI’s supervision. We will provide the project manager with an implementation manual (online supplemental file 1) hosted on Research Electronic Data Capture (REDCap) software, which comprises detailed guidance regarding when and how to implement each of the five CHARMER intervention components. REDCap will also include a checklist of implementation milestones for each intervention component to enable project managers, PIs, Health Innovation East and the research team to monitor implementation progress.

The Health Innovation Network^10^ will support project managers and PIs throughout the implementation period. This will include scheduling a set of meetings followed by weekly meetings to discuss any challenges or queries about implementation, and quarterly Community of Practice meetings where sites in previous steps can support new sites in navigating implementation issues. As the Health Innovation Network is the arm of NHS England that supports the implementation of innovation across the NHS, the CHARMER implementation support provided is nationally scalable beyond the trial environment, should CHARMER be found effective and cost-effective.

### Process evaluation research team

The process evaluation team comprises methodologists (JMM-K, JT, IK, BA, EMB-F, ABC), pharmacists (DB, SS, DPA, DJW, CELJ), a geriatrician (VLK) and patient and public involvement (PPI) members. The researchers undertaking the interviews for the process evaluation were not involved in implementation processes to enable participants to provide their perspectives to people neutral to the intervention implementation.

### Process evaluation overview

We will conduct a convergent parallel design process evaluation^11^ underpinned by Normalisation Process Theory (NPT) and the Theoretical Domains Framework (TDF). This aims to understand how the CHARMER intervention was implemented and received, and the mechanisms of action by which it exerts its effects on geriatricians’ and pharmacists’ deprescribing behaviour. The objectives are to:

- Describe the fidelity of CHARMER intervention implementation, including any adaptations.
- Understand how the intervention addresses the intended barriers and enablers to proactive deprescribing and any unintended consequences.
- Explore contextual factors that influence intervention implementation and effectiveness.
- Explore the acceptability of the intervention by different stakeholders.
- Explore the perceived effect of the intervention.
- Explore the acceptability and feasibility of trial processes.

**Figure 3:**
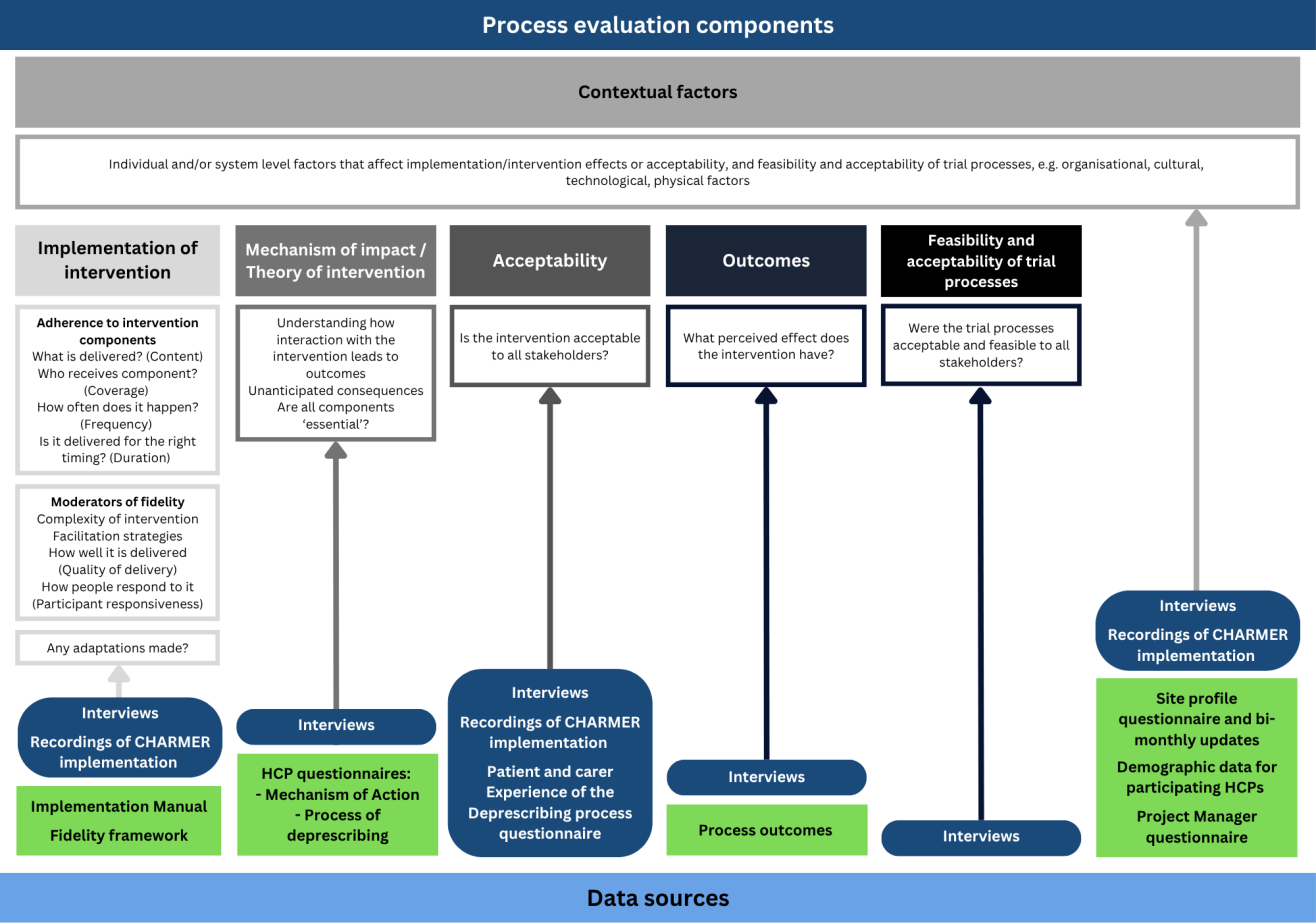
Components of the CHARMER process evaluation. Green refers to quantitative data, blue refers to qualitative data. CHARMER, CompreHensive geriAtRician-led MEdication Review; HCPs, healthcare professionals.

Figure 3 provides an overview of the different components of the process evaluation. This summarises how each data source will be used to understand how the intervention is implemented, contextual factors present at sites that influence the intervention, mechanisms of impact, acceptability and outcomes of the intervention, and acceptability and feasibility of trial processes. We have described these data sources in more detail within the data sources section.

### Participants

### Hospital and primary care staff participants

We will consent to process evaluation data for all:

- Principal investigators.
- Research delivery staff involved in patient/carer recruitment and data collection.
- Project managers leading local CHARMER implementation.
- Geriatricians and pharmacists who are intended to receive the CHARMER intervention.

To explore the effect of CHARMER on primary care, we will recruit general practitioners and primary care pharmacists from general practices who have at least one patient recruited in CHARMER during the intervention enhanced data collection period and who had a medicine(s) stopped. Hospitals will send a letter to all recruited patients’ general practice notifying them of their participation in the CHARMER trial. A second letter will be posted or emailed by the CHARMER team to the primary care practice requesting an interview.

### Patient and carer participants

Patients under the care of a geriatrician recruited to CHARMER who have a medicine(s) stopped during the intervention enhanced data collection period will be recruited. For patients who cannot provide consent due to lack of capacity, their family member(s) or care staff member for those patients residing in a care home will be approached to provide consent to participate.

### Data sources

#### Recordings of CHARMER implementation (all intervention sites)

**Aim**: *to understand what was implemented at each site, how the intervention was received and by whom, and any adaptations made.*

All sites will be asked to audio-visually record the implementation of three of the five intervention components: action plan launch, pharmacist workshop and geriatrician workshop. The development and review of benchmarking reports and weekly briefings will not be recorded, as it is likely that patient data would be discussed. We will explore these two intervention components during interviews with hospital staff participants.

#### Implementation manual (all intervention sites)

**Aim:** *to understand what was implemented at each site and by whom it was received.*

To enable the implementation team to monitor what is implemented at each site in real time, implementation manuals within REDCap (online supplemental file 1) will be completed by the project managers (see figure 3, ‘Implementation of intervention’). The implementation team (BA, CELJ) will review submitted documents to check for completeness and accuracy and follow-up with the project manager to address any gaps. The reported completeness of intervention implementation activities may also inform refinement of implementation support for future steps.

#### Fidelity framework

**Aim**: *to understand how the intervention components were implemented, including any adaptations and how the components were received.*

To understand how the intervention components are delivered and received, and whether each component is essential for any outcomes achieved, requires measurement of the CHARMER intervention implementation. We selected the Conceptual Framework for Implementation Fidelity, as this framework, when operationalised provides a practical way to consistently measure implementation fidelity. To ensure consistent and objective evaluation of how each CHARMER intervention component is implemented and received across sites, we will refine the fidelity framework that was developed within the feasibility study.^6^ The fidelity framework was guided by the Conceptual Framework for Implementation Fidelity^12^ and we will apply their definitions to inform a scoring system to enable detailed reporting on:

- Adherence: including content, frequency, duration and coverage of each component.
- Moderators of fidelity: such as component complexity, facilitation strategies, delivery quality and participant responsiveness.

From adherence data, we will estimate the dose of each intervention component delivered. The evaluation will include both quantitative fidelity scores and qualitative insights to explain site-level variations.

### Process of Deprescribing Practitioner Questionnaire (all intervention sites)

**Aim**: *to understand whether CHARMER addresses the intended barriers and enablers of proactive deprescribing and the steps practitioners undertake when proactively deprescribing medicines.*

The Process of Deprescribing Practitioner Questionnaire (online supplemental file 2; figure 3, ‘Mechanism of impact/theory of intervention’) is a self-reported measure of healthcare professionals’ proactive deprescribing behaviour. The questionnaire captures the extent to which the 17 activities that make up the four proactive deprescribing steps are enacted by intervention recipients. The questionnaire was developed and validated with 236 healthcare professionals and researchers from 25 countries.^13^ Responses to the 19 questionnaire items are invited on a 5-point Likert scale from ‘never’ undertake the proactive deprescribing activity to ‘always’.

All participating geriatricians and pharmacists will be invited to complete the Process of Deprescribing Practitioner Questionnaire twice; once before receiving the CHARMER intervention and once at 6 weeks into the intervention period. This is to allow participating geriatricians and pharmacists to receive the action plan and relevant workshop, and for them to receive some weekly benchmarking reports and have the opportunity to attend some weekly briefings where deprescribing opportunities are discussed.

### Deprescribing Behaviour Change Mechanism of Action questionnaire (all intervention sites)

The Deprescribing Behaviour Change Mechanism of Action (DBCMoA) questionnaire^6^ (see figure 3, ‘Mechanism of impact/theory of intervention’; and online supplemental file 2) is a self-reported measure of the extent to which the behavioural determinants of proactive deprescribing in hospital are experienced by healthcare professional respondents. The development of the DBCMoA questionnaire is comprehensively reported elsewhere, and a summary is provided.^6^

Questionnaire items are validated measures of behavioural determinants derived from the Organisational Readiness to Change Assessment (ORCA) tool.^14^ Validated measures were selected by mapping healthcare professionals’ behavioural determinants of proactive deprescribing in hospital to the Consolidated Framework for Implementation Research (CFIR),^15^ and then through the linkage between CFIR domains and ORCA measures, selecting relevant validated measures for the mapped CFIR domains. Measures were then adapted to the context of proactive deprescribing in hospitals while retaining their measurement of the underlying mechanism of action of behaviour change.^6^ The DBCMoA comprises behavioural determinants intended to be addressed by the CHARMER intervention and other potential behavioural determinants. This will permit exploration of whether the CHARMER intervention operates via the intended behavioural mechanisms. Responses to the 11 questionnaire items are invited on a 5-point Likert scale to indicate the extent to which they are experiencing different determinants of proactively deprescribing from ‘strongly disagree’ to ‘strongly agree’.

### Patient and carer Experience of the Deprescribing Process Questionnaire (all intervention sites)

**Aim**: *to understand how patients and their carers found the process of a medicine(s) being stopped in hospital.*

The Patient Evaluation of the Deprescribing Process Questionnaire (PED-Q) is a validated Patient Reported Experience Measure^16^ designed to capture the patient and carer experience of the proactive deprescribing process under the care of geriatricians and pharmacists who received the CHARMER intervention.^13^ The PED-Q (online supplemental file 3) comprises eight items that measure a patient’s or carer’s experience of a healthcare professional undertaking the proactive deprescribing activities that can be experienced by a patient or carer. For each item, responses are invited on a 5-point Likert scale ranging from ‘very dissatisfied’ to ‘very satisfied’ with the proactive deprescribing activity received.

Patients or their carers will be asked to complete the questionnaire within 2 weeks of hospital discharge, either by posting a completed hard copy to us, or by completing it over the telephone.

### Site profile questionnaire and bi-monthly updates (all sites)

**Aim**: *to characterise sites, identify any changes during the study period and understand contextual factors that may influence the intervention.*

The PI at each participating hospital site will identify their OPM study ward(s) prior to the trial beginning and confirm the number of beds.

We have developed a site profile questionnaire (see online supplemental file 2 and figure 3 ‘Contextual factors’) to capture resourcing at sites, the electronic records system used and whether any activities related to medicines optimisation or deprescribing, apart from CHARMER, are happening at the hospital. All PIs will be invited to complete the site profile questionnaire at the beginning of the trial and then review their responses for any changes immediately after implementation is complete, and approximately 3 months before the trial ends.

Additionally, we will email PIs bi-monthly to capture changes to the study ward(s), for example, due to infection or change of ward type or change in bed numbers, changes to participating geriatricians and pharmacists, any repeat delivery of the CHARMER intervention for new geriatricians and pharmacists, and whether briefings and benchmarking reports are happening as intended during the intervention period.

### Demographic questionnaires (all sites)

**Aim**: *to capture contextual information that may help explain the intervention’s effects.*

All participating geriatricians and pharmacists will complete a demographics questionnaire at the beginning of the trial. This will capture their age, gender, ethnicity, role, current grade/band and the number of years in their current role. This will allow us to understand who received the intervention and explain any differences in receipt and enactment of the intervention (see figure 3, ‘Contextual factors’).

### Project manager questionnaire (all intervention sites)

**Aim**: *to explore barriers and enablers for implementing CHARMER outside the trial parameters.*

Project managers at each site will be asked to complete an electronic questionnaire (online supplemental file 2) asking them about barriers and enablers to implementing the CHARMER intervention at their hospital. The questionnaire will be sent 4–6 weeks post implementation to allow sites to have implemented benchmarking reports and briefings.

### Semi-structured interviews (all sites)

#### Aims

- To understand implementation, how the intervention was received and any perceived impact of the intervention and any contextual factors that may influence this (PIs, project managers and participating geriatricians and pharmacists—all intervention sites).
- To understand geriatrician and pharmacist recruitment and participation (PI, project managers and participating geriatricians and pharmacists—all intervention sites).
- To understand patient/carer recruitment and data collection processes (research delivery teams—all sites).
- To explore patients’ and their carers’ experience of having a medicine(s) stopped in hospital (patients and carers—all intervention sites).
- To understand the impact of CHARMER and hospital deprescribing in general on primary care (primary care).

### Interview sampling and procedures

We will use topic guides refined from the CHARMER feasibility study (online supplemental file 4) to fulfil the aims. Sampling will be undertaken to ensure we hear from a wide range of patients, carers and staff and we will continue interviewing if new data arise in interviews. Interviews will be undertaken by three qualitative researchers (JMM-K, EMB-F, and Amanda Edmondson) with the following participants:

- All project managers (45 min; 6 weeks post implementation).
- All PIs (60 min; towards the end of trial).
- 40–60 geriatricians and pharmacists (45 min, towards the end of the trial).
  - Sampling to achieve maximum variation in barriers and enablers to deprescribing at baseline (see the Deprescribing Behaviour Change Mechanism of Action Questionnaire section), length of professional experience and demographics.
- Research delivery (30 min; after the second patient recruitment period).
  - One person nominated by the site.
- 20–30 patients and carers (15–20 min; within 2 weeks of discharge for those recruited during the intervention period).
  - Sampling to achieve variation in ethnicity, age, gender and medicines stopped.
- 10–15 prescribing primary care practitioners (30 min; ≥3 months after the patient is discharged from the hospital).
  - Sampling to achieve variation in role (eg, general practitioner, pharmacist, nurse practitioner) across the country.

Interviews will be offered via Microsoft Teams or telephone. All interviews will be digitally recorded, transcribed and anonymised. To support participation and inclusivity, patients and carers can choose to provide their feedback about having a medicine(s) stopped in hospital by completing a written form as an alternative to an interview.

### Process outcomes (all intervention sites)

**Aim**: *to explore the impact of the CHARMER intervention.*

We will report process outcomes, including the number of regularly prescribed medicines stopped on the study wards, the number of pre-admission medicines with dosage reduced, number of medicines that are restarted after discharge from the hospital.

### Analysis

#### Quantitative analysis

##### Fidelity data

Quantitative data from the fidelity framework will be analysed descriptively. We will synthesise findings and report these for each intervention component in a tabular format to enable us to explain differences in the CHARMER intervention effectiveness between sites.

##### Site profile questionnaire and PI questionnaires

These will be analysed descriptively and summarised for sites overall and by their allocation sequence in the trial (step).

##### process of deprescribing practitioner and deprescribing behaviour change mechanism of action questionnaires

We will analyse the responses for both questionnaires descriptively (median, IQRs) to understand differences in responses across sites and between individuals. We will compare responses before and after the intervention to determine whether self-reported proactive deprescribing behaviour has changed and the behavioural mechanism(s) by which the reported change has occurred. These data will also be included in the effectiveness analysis. The analysis conducted will be a Mann-Whitney test of the change in the response between preintervention and postintervention.

##### patient and carer experience of the deprescribing process

We will analyse the responses for both questionnaires descriptively (median, IQRs) to understand differences in responses across sites and between individuals.

##### Process outcomes

These data will be analysed descriptively within the main effectiveness analysis of the CHARMER trial (manuscript submitted). These analyses will also be used within the process evaluation to enable explanation of the impact of the CHARMER intervention on geriatricians’ and pharmacists’ deprescribing activity at each site.

### Qualitative analysis

#### Thematic analysis and coding

We will use thematic analysis^17^ guided by a coding dictionary and supported by NVivo. During the feasibility study, the data were analysed using inductive reflexive thematic analysis, enabling the development of codes related to barriers and enablers to deprescribing and implementation of the CHARMER intervention, and the process of recruitment and participation in the trial. These codes will be included within the dictionary and refined, with further codes developed as the data are thematically analysed. Two researchers will undertake the thematic analysis and the dictionary will enable consistency of data analysis.

To ensure the trustworthiness of the data analysis, we will incorporate a number of approaches. Weekly process evaluation meetings throughout the data collection and analysis periods, including intervention, clinical and methodological expertise, and regular input from PPI members, will be undertaken. These meetings will be used to discuss the data and review supporting quotes underlying codes and to address queries that arise during the analysis to increase rigour and consistency within the analysis. This process will ensure the analysis is credible. Both coders (JMM-K, EMB-F) will also maintain reflexive diaries about their thoughts on the data and any biases or focus that they are aware of when coding to ensure confirmability. Their background and how this has influenced their data collection and analysis will be recorded to capture and report reflexivity and the role this has played in the analysis process. We will maintain an audit trail of all research steps, decisions and methodological changes made during the analysis to ensure dependability of the analysis. Finally, we will ensure we report data across all sites, including contextual aspects, to enable transferability of the findings.

### Artificial intelligence

A novel approach to our analysis will be the use of Copilot, an artificial intelligence (AI) software as a team member, with oversight by the research team, to provide another perspective on the data analysis.^18 19^ We will use several test transcripts to see how Copilot performs and aligns with the other team members’ coding. We will document any discrepancies between AI and human coding to refine the process. Assuming Copilot analyses transcripts aligning with the team’s process and approach, it will independently analyse some of the transcripts, with checking of the coding undertaken by members of the research team.

### Mapping to theory frameworks

We will use NPT^20 21^ to help understand intervention implementation and the TDF^22^ to help understand how the intervention exerted its effects. NPT is an implementation theory and application of NPT within the CHARMER definitive trial process evaluation will provide a lens through which to understand and explain why the CHARMER intervention was/was not implemented. This, in turn, will provide learning to inform a future national strategy for intervention adoption and implementation across NHS hospitals in England. We will map codes to NPT and will also explore key contextual aspects that influenced CHARMER and proactive deprescribing at sites. Mapping will be undertaken by two researchers (JMM-K, EMB-F) as well as by Copilot.

The CHARMER intervention is underpinned by the TDF, which is a synthesis of behavioural science theories organised into 14 domains, for example, ‘Knowledge’, ‘Beliefs about consequences’ and ‘Social/professional role and identity’.^22^ Application of TDF within the CHARMER definitive trial process evaluation permits exploration of the behavioural mechanisms by which the intervention produces its effects and whether this happened via the intended behavioural mechanisms (ie, TDF domains) and whether any other barriers or enablers are unintentionally addressed by CHARMER. We will explore this by mapping codes describing barriers and enablers to deprescribing to the TDF.

### Synthesis and triangulation

We will use the analysis process to understand what happened at each site to enable an explanation of the effects of the CHARMER intervention. Triangulation will involve data from each qualitative and quantitative component of the study. We will visually present these data in tables and figures to allow us to identify where there is agreement or disagreement between findings from different data components and thus identify how the intervention may need to be modified. The triangulation process will be decided iteratively based on what we find when analysing separate data sources to enable us to explore this in more detail. Based on learning from this process evaluation, we will refine the CHARMER intervention logic model.

### Patient and public involvement

We have five PPI team members who have provided input into discussions and decisions relating to the process evaluation study design. This includes involvement in the topic guides, input into the patient-facing documents and planning around how to effectively obtain feedback from older people participating in CHARMER. The PPI team will participate in discussions about the process evaluation data and its interpretation.

### Ethics and dissemination

Our study has received ethical approval (REC ID: 23/WA/0184) and study approval from the Health Research Authority (Project ID: 323504). We also sought Capability & Capacity confirmation from sites prior to the trial beginning. We will follow the Guide to Disseminating Research (GuiDiR)^23^ when disseminating findings from the process evaluation.

## Supplementary material

10.1136/bmjopen-2025-111152 online supplemental file 1

10.1136/bmjopen-2025-111152 online supplemental file 2

10.1136/bmjopen-2025-111152 online supplemental file 3

10.1136/bmjopen-2025-111152 online supplemental file 4

## References

[1] Gallagher P, Lang PO, Cherubini A (2011). Prevalence of potentially inappropriate prescribing in an acutely ill population of older patients admitted to six European hospitals. Eur J Clin Pharmacol.

[2] Organisation WH (2017). Global patient safety challenge: medication without harm.

[3] Scott S, Clark A, Farrow C (2018). Deprescribing admission medication at a UK teaching hospital; a report on quantity and nature of activity. Int J Clin Pharm.

[4] Scott S, Twigg MJ, Clark A (2019). Development of a hospital deprescribing implementation framework: A focus group study with geriatricians and pharmacists. Age Ageing.

[5] Scott S, Atkins B, Kellar I (2023). Co-design of a behaviour change intervention to equip geriatricians and pharmacists to proactively deprescribe medicines that are no longer needed or are risky to continue in hospital. Res Social Adm Pharm.

[6] Scott S, Atkins B, Martin-Kerry JM (2023). CompreHensive geriAtRician-led MEdication Review (CHARMER): protocol for a feasibility study of a hospital deprescribing behaviour change intervention. BMJ Open.

[7] Scott S, Martin-Kerry J, Pritchard M (2026). The feasibility of implementing a hospital deprescribing behaviour change intervention and undertaking trial processes: A mixed methods evaluation. Res Social Adm Pharm.

[8] Moore GF, Audrey S, Barker M (2015). Process evaluation of complex interventions: Medical Research Council guidance. BMJ.

[9] Health Innovation Network.

[10] Wright DJ, Alldred DP, Scott S (2026). Evaluation of the CompreHensive geriAtRician-led MEdication Review (CHARMER) deprescribing intervention in hospital: protocol for a cluster randomised stepped-wedge trial. BMJ Open.

[11] Creswell J, Plan Clark V (2018). Designing and Conducting Mixed Methods Research.

[12] Carroll C, Patterson M, Wood S (2007). A conceptual framework for implementation fidelity. Implement Sci.

[13] Scott S, Buac N, Bhattacharya D (2024). An Internationally Derived Process of Healthcare Professionals’ Proactive Deprescribing Steps and Constituent Activities. Pharmacy (Basel).

[14] Helfrich CD, Li Y-F, Sharp ND (2009). Organizational readiness to change assessment (ORCA): development of an instrument based on the Promoting Action on Research in Health Services (PARIHS) framework. Implement Sci.

[15] Damschroder LJ, Reardon CM, Widerquist MAO (2022). The updated Consolidated Framework for Implementation Research based on user feedback. Implement Sci.

[16] COSMIN (2018). COSMIN methodology for systematic reviews of patient‐reported outcome measures (proms).

[17] Miles MB, Huberman AM (1994). Qualitative Data Analysis: An Expanded Sourcebook.

[18] Morgan DL (2023). Exploring the Use of Artificial Intelligence for Qualitative Data Analysis: The Case of ChatGPT. Int J Qual Methods.

[19] Davison RM, Chughtai H, Nielsen P (2024). The ethics of using generative AI for qualitative data analysis. *Information Systems Journal*.

[20] May CR, Albers B, Bracher M (2022). Translational framework for implementation evaluation and research: a normalisation process theory coding manual for qualitative research and instrument development. Implement Sci.

[21] Murray E, Treweek S, Pope C (2010). Normalisation process theory: a framework for developing, evaluating and implementing complex interventions. BMC Med.

[22] Atkins L, Francis J, Islam R (2017). A guide to using the Theoretical Domains Framework of behaviour change to investigate implementation problems. Implement Sci.

[23] Scott S, Atkins B, D’Costa T (2024). Development of the Guide to Disseminating Research (GuiDiR): A consolidated framework. Res Social Adm Pharm.

