# Supplementary material for: Implementation of a hospital deprescribing behaviour change intervention, the CompreHensive geriAtRician-led MEdication Review (CHARMER) trial: a process evaluation protocol
Source: BMJ Open. 2026 Jun 2;16(6):e111152. doi: 10.1136/bmjopen-2025-111152 (PMC13239467; doi:10.1136/bmjopen-2025-111152)
Supplement: online supplemental file 1 [file bmjopen-16-6-s001.docx]

**CHARMER Implementation Manual**

*Content to be embedded into REDCap*

**Introductory text for landing page:**

This manual will support the Project Manager to implement the CHARMER intervention, ensuring that all five components are delivered as intended. It will also ensure that all participating geriatricians and pharmacists receive all relevant components.

The manual incorporates a checklist for each component to help you monitor the progress of CHARMER implementation at your hospital. The Project Manager is required to **complete all actions** in the manual and record progress in the checklists. Your answers will be saved on the REDCap system and you can log in to continue/edit your submissions over the next 14 weeks.

**Question on landing page:**

All geriatricians and pharmacists providing care on the study ward(s) **MUST** have completed the consent form and baseline questionnaires **BEFORE** they receive the CHARMER intervention.

**Has the PI confirmed that all of these geriatricians and pharmacists have completed the consent form and baseline questionnaires?**

Yes

No

Please introduce yourself to the participating geriatricians and pharmacists and explain your role in supporting implementation. We have provided a pre-prepared email template – feel free to edit this or write your own.

**Have you introduced yourself to all participating geriatricians and pharmacists? Please explain how you did this below (e.g., via email using our template, or a different method?)**

Free text

**Link to each component:**

1. Action plan for proactive deprescribing
2. Benchmarking reports on proactive deprescribing activities
3. Weekly pharmacist and geriatrician proactive deprescribing briefings
4. Workshop for pharmacists
5. Workshop for geriatricians

# 1. Action plan for proactive deprescribing

**Overview of component**

This intervention component comprises the development and launch of your hospital’s proactive deprescribing action plan. An action plan outlines a strategy to achieving a specific goal. In this instance, the goal is to increase proactive deprescribing within your hospital. The strategy to achieve this goal is to implement the remaining four CHARMER intervention components.

We have provided a partially-populated action plan template (see below) to help you identify what resources are required to implement the CHARMER intervention. Upon completion, the action plan needs to be authorised and launched in order to highlight that proactive deprescribing is a priority in your hospital.

**When this needs to happen**

You need to have developed and launched your hospital’s proactive deprescribing action plan by **no later than 28 June 2024.**

The action plan launch **must** be recorded – refer to our guidance [**hyperlink**] and please arrange how you will do this with the CHARMER team or Health Innovation East (HIE) when you get to this stage.

**What you need to do**

| **Implementation phase (1 May 2024 – 31 July 2024)** | |
| --- | --- |
| Download and complete the action plan template [**hyperlink**]. Alternatively, you can transfer the content to your hospital’s own action plan template. | Box to automatically populate with the date the action plan was downloaded |
| Get the action plan authorised. The action plan template provides guidance on how, and by whom, you may get your action plan authorised. | Upload the authorised action plan here:  Upload box |
| Launch the action plan in your hospital **by no later than 28 June 2024.** | **Please provide details on the activities you have taken to prepare for the launch of your hospital’s action plan.**  E.g., Have you booked a room for the group meeting (if necessary)? Have you organised a facilitator?  Free text |
| The action plan launch event must be audio-visually recorded so that we can review attendee engagement.  Look over the recording guidance and discuss this with a CHARMER/HIE team member to ensure you have a plan in place of how you will record attendees and how you will send the recording to CHARMER for review. | **Have you discussed with the CHARMER/HIE team how you will record your action plan launch event?**  Yes  No  **What are your plans for recording the launch?**  Free text |
| Familiarise yourself with the reporting information (see below) you need to capture and submit for this intervention component. | **Are you clear on what reporting information must be collected and submitted for this component?**  Yes  No |

| **Reporting information (to be submitted after the action plan launch, and by no later than 9 August 2024)** | |
| --- | --- |
| What date was the action plan launched? | Date/calendar |
| What active launch event activities did you undertake? Tick all that apply. | **A launch event to announce the new proactive deprescribing initiative at:**  In person or virtual launch meeting/presentation  Grand Round  Departmental meeting for Older People’s Medicine  Departmental meeting for Pharmacy  Staff briefings  Other (Please specify): |
| Did the launch event take place in-person, online or hybrid? | In-person  Online  Hybrid |
| Who facilitated/led the launch event (please provide role only)? | Free text box |
| What passive launch activities for the action plan did you undertake? Tick all that apply. | Proactive deprescribing screensavers in Older People’s Medicine ward(s)  E-mail to relevant staff, including geriatricians and pharmacists  e-bulletins or newsletters circulated within the trust  Circulated webinar  Other (Please specify): |
| What launch materials did you use? | Provided by the CHARMER team:  Screensaver  PowerPoint slides  Email to senior staff  Please upload any resources you used, that were not provided by the research team, here (e.g., emails or bulletins):  Upload box (x5) |
| Names of participating geriatricians who attended the launch event: | Free text |
| Names of participating pharmacists who attended the launch event: | Free text |
| Please upload the attendance register here (please provide roles only): | Upload |
| To the best of your knowledge, was the launch event recorded? | Yes  No |
| How many person hours do you think were spent *developing* the action plan? | Free text box |
| How many person hours do you think were spent *launching* the action plan? | Free text box |
| Is there anything you would like to note about the implementation of the proactive deprescribing action plan? For example, please note anything you found either particularly challenging or straightforward. | Free text box |

**Resources** [**hyperlinks**]

- Action plan launch – template slides
- Action plan launch - email template to relevant staff
- Action plan launch - screensaver
- Attendance register
- Guidance on how to record action plan

# 2. Benchmarking reports on proactive deprescribing activities

**Overview of component**

This intervention component comprises weekly reports on proactive deprescribing activities. The purpose of this intervention component is to incentivise pharmacists and geriatricians to proactively deprescribe by benchmarking their proactive deprescribing progress. The weekly benchmarking reports will capture four elements:

1. Number of beds on the study ward(s)
2. Number of preadmission medicines
3. Number of preadmissions medicines stopped
4. Number of deprescribing discussions between a healthcare professional and patient

**When this needs to happen**

The benchmarking reports will be produced and reviewed weekly throughout the active intervention phase (**starting 1 August 2024**). Preparation needs to start **immediately during the implementation phase** to ensure relevant team members are familiar with how to:

- Record, extract and submit the required data to the CHARMER team
- Share and review the benchmarking reports.

**What you need to do**

| **Implementation phase (1 May 2024 – 31 July 2024)** | |
| --- | --- |
| Download and familiarise yourself with the benchmarking reports guidance document [**hyperlink**]. This document provides an overview of what proactive deprescribing data the benchmarking reports will capture, who is responsible for recording, extracting, and submitting the data, and who is responsible for sharing and reviewing the benchmarking reports. | Box to automatically populate with the date the document was downloaded. |
| The Project Manager and person responsible for submitting the benchmarking data will need to attend a 30-minute planning meeting with Luke Natali to sign off plans for how the required data will be recorded, extracted and submitted. The planning meeting needs to take place between **20-31 May 2024.**  Please agree a suitable date and time for the 30-minute CHARMER database meeting with the person responsible for submitting the benchmarking data. The team will then be in touch to confirm your meeting time. | **What date/time would you like to attend the meeting with Luke?**  Free text box |
| **Please complete the following actions *BEFORE* your planning meeting. Note: These are provisional plans – you can edit and resubmit your answers after your planning meeting.** | |
| **Action 1: Develop a strategy to ensure that each deprescribing discussion between a healthcare professional and patient is recorded.** | |
| Identify a member of the **electronic prescribing/electronic health records** **team.** Work with them and the geriatricians and pharmacists participating in CHARMER to develop a strategy to ensure that each deprescribing discussion between a healthcare professional and patient is recorded. For example, you may want to add a deprescribing discussion check box into your system so that clinicians can easily record any discussions. | **What is your strategy for recording the number of deprescribing discussions between a healthcare professional and patient?** |
| **Action 2: Develop a strategy for extracting and submitting the required data** | |
| Identify a member of the **electronic prescribing/electronic health records team** (note: this may be the same team member involved above). This person will be responsible for extracting and submitting the required data into the CHARMER database each week throughout the active intervention phase.  As noted in the guidance document, this data will be requested via email every Monday throughout the active intervention phase. The team member will need to extract and submit the data for the previous week (Monday-Sunday). | **Who have you identified to extract and submit the required data throughout the active intervention phase?**  Name:  Role:  Email: |
| The above team member will need to extract and submit the following data:   1. **Number of beds on the study ward(s)**   The total number of beds on the study ward(s). This should remain the same week to week and will be pre-filled for your ease after the first data submission. For example, on a hypothetical ward with a total capacity of 50 beds, you would submit 50. | **Please briefly detail below how this data will be extracted.** |
| 1. **Number of preadmission medicines**   Across all patients on the study ward(s), please combine the number of medicines (including PRN/when required) each patient was taking **before** coming into hospital and **before** any changes have been made (i.e., patient’s drug history/medicines reconciliation medicines). For example, on a hypothetical ward with 40 patients prescribed a combined total of 200 preadmission medicines across all patients, you would submit 200. | **Please briefly detail below how this data will be extracted.** |
| 1. **Number of preadmissions medicines stopped**   Across all patients on the study ward(s), please combine the number of preadmission medicines that were stopped. For example, on a hypothetical ward with 40 patients with a combined total of 50 preadmission medicines stopped, you would submit 50. | **Please briefly detail below how this data will be extracted.** |
| 1. **Number of deprescribing discussions between a healthcare professional and patient**   Across all patients on the study ward(s), please combine the number of discussions between a healthcare professional and patient or their representative about potentially stopping preadmission medicine(s), as defined above. | **Please briefly detail below how this data will be extracted.** |
| **Please complete the following actions *after* your planning meeting.** | |
| **What date did you attend the planning meeting?**  Date/calendar | |
| **Action 3: Submit test data and confirm receipt of test benchmarking report**  Team member responsible for data extraction and submission to submit test data into CHARMER database between **3-14 June 2024**.  You (Project Manager) will receive a test benchmarking report triggered by the submission of the test data. Please confirm you have received this and complete the embedded survey. | **Has the team member responsible for submitting benchmarking data received a test data submission survey and submitted their test data?**  Yes  No  **Were there any issues extracting or submitting the data?**  Yes  No  If yes…  **Please describe the issues:**  Free text box  **Have you (Project Manager) received the test benchmarking report (please check junk folder)?**  Yes  No  **Did you have any issues accessing the report?**  Yes  No  If yes…  **Please describe the issues:**  Free text box |
| **Action 4: Communicate requirement to record the number of deprescribing discussions between a healthcare professional and patient starting 1 August 2024**  It is the responsibility of any healthcare professional (including junior doctors) who has a deprescribing discussion to record this throughout the active intervention phase. Inform all relevant team members on the study ward(s) of the requirement to record the number of deprescribing discussions via the agreed method. For example, if you have added a deprescribing discussion checkbox into your system, explain this to all relevant team members. | **Have all relevant team members on the study ward(s) been informed of the requirement to record the number of deprescribing discussions between a healthcare professional and patient starting 1 August 2024?**  Yes  No  **How did you communicate this requirement?**  Free text |
| **Action 5: Develop strategy for reviewing the benchmarking reports**  Throughout the active intervention phase, all geriatricians and pharmacists participating in CHARMER will need to review the benchmarking reports. As detailed in the benchmarking guidance document, they will receive the reports via email every **week starting w/c 12 August 2024.** They need to review the reports with their colleagues and may do this:   - During their weekly briefings (planned or ad hoc) - During another planned or ad hoc meeting, such as a ward round   During the meetings, the pharmacists and geriatricians can view the reports via their email (i.e., on their phone/a computer). Alternatively, they may prefer to nominate a team member(s) to print the report each week. A copy of the report could be displayed on the study ward(s) for geriatricians and pharmacists to review during their briefings/meetings. If you cannot display the reports on the study ward(s), the printed reports could instead be shared by the nominated team member(s) during their weekly briefings/meetings.  Either way, a team member must confirm the benchmarking report has been reviewed each week by completing the embedded survey in the report.  Work with participating pharmacists and geriatricians to plan how the reports will be reviewed. We recommend you do this during your weekly briefing planning meeting. | **What is the agreed plan for reviewing the benchmarking reports?**  Free text |
| Familiarise yourself with the reporting information (see below) you need to capture and submit for this intervention component. | **Are you clear on what reporting information must be collected and submitted for this component?**  Yes  No |

| **Active intervention phase (starting 1 August 2024)** | |
| --- | --- |
| **First day of active intervention phase:** Remind all team members involved in the benchmarking report process that the active intervention phase (and thus their role/responsibility in recording the required data, extracting/submitting the required data, and/or reviewing the reports) has begun. Reminder emails to be sent include:   - Reminder to the team member responsible for extracting and submitting the required data that their role starts now. - Reminder to all relevant team members on the study ward(s) of the requirement to record the number of deprescribing discussions via the agreed method (it is the responsibility of any healthcare professional who has a deprescribing discussion to record this – including junior doctors). - Reminder to all geriatricians and pharmacists participating in CHARMER of the requirement to review the benchmarking reports.   You may want to use our pre-prepared reminder email template. | **Have all relevant team members been reminded that the active intervention phase – and their responsibility/role in the benchmarking report process – has begun?**  Yes  No |
| Check that the first week of benchmarking data has been extracted and submitted. | **Has the first week of benchmarking data been extracted and submitted?**  Yes  No  **Were there any issues or challenges with extracting or submitting the benchmarking data?**  Yes  No  **If yes, please describe the issues below:**  Free text |

| **Reporting information (to be submitted by no later than 9 August 2024)** | |
| --- | --- |
| How many person hours do you think were spent planning the benchmarking report process? | Free text box |
| Were any other team members involved in the benchmarking process (e.g., did the IT department help with data extraction plans)? Please provide roles only. | Free text box |
| How long was spent extracting and submitting the benchmarking data? (Please discuss with delegated team member who took on this role) | Free text box |
| Is there anything you would like to note about the implementation of the benchmarking reports? For example, please note anything you found either particularly challenging or straightforward. | Free text box |

**Resources** [**hyperlinks**]

- Pre-prepared reminder email template to geriatricians and pharmacists
- Guidance on how to submit benchmarking data/schedule

# 3. Weekly pharmacist and geriatrician proactive deprescribing briefings

**Overview of component**

This intervention component comprises weekly in-person briefings between participating pharmacists and geriatricians to provide protected time for them to discuss opportunities for proactive deprescribing on study ward(s).

**When this needs to happen**

The weekly briefings will take place throughout the active intervention phase (**starting 1 August 2024**). You need to formulate a plan **during the implementation phase** for when and how the weekly briefings will occur so that all pharmacists and geriatricians taking part in the CHARMER trial are ready to start on this date.

**What you need to do**

| **Implementation phase (1 May 2024 – 31 July 2024)** | |
| --- | --- |
| Familiarise yourself with the weekly briefings overview and planning template [**hyperlink**]. You will need to complete this during your planning meeting with participating geriatricians and pharmacists (see below). | **Date planning template downloaded:**  Automatically populate  **Do you have any questions about the weekly briefings and what is required?**  Free text |
| Organise for the participating pharmacists and geriatricians to attend a planning meeting to agree how they will conduct the weekly briefings during the active intervention phase. This meeting must take place at least **4 weeks before** the active intervention phase starts (by no later than **5 July 2024**). Include the following resources in your communications:   - Weekly briefings overview and planning template - Deprescribing guide sheet for pharmacists   You may want to use our pre-prepared email template [**hyperlink**].  During the planning meeting, work with pharmacists and geriatricians to identify their preferred method for the weekly briefings. For example, they may want to select a particular time and day each week, or they may prefer ad-hoc meetings. Please record your plans on the weekly briefings overview and planning template [**hyperlink**] and upload this once complete. Alternatively, you can submit your plans directly into the free text box. | **Have all pharmacists and geriatricians participating in CHARMER attended a planning meeting for the weekly briefings?**  Yes  No  **You MUST complete at least one of the following:**   - **Upload the completed weekly briefing planning template here:**   Upload  **OR detail your plans below:**  *Please briefly explain your plans for conducting the weekly briefings, including format (one-to-one or group briefings, ad-hoc or scheduled) and expected number of briefings per week.*  Free text box |
| During the planning meeting, remind everyone that they are required to review the weekly benchmarking reports and, if you have not already done so, establish their preferences for how they want to do this. Review the benchmarking reports guidance document for more information.  We also suggest that you show colleagues your test benchmarking report/the embedded survey during this planning meeting so that they are familiar with the format/process. | **Have all participating geriatricians and pharmacists been reminded of the requirement to review the benchmarking reports and how they may do this?**  Yes  No |
| Familiarise yourself with the reporting information (see below) you need to capture and submit for this intervention component. | **Are you clear on what reporting information must be collected and submitted for this component?**  Yes  No |

| **Active intervention phase (starting 1 August 2024)** | |
| --- | --- |
| **First day of active intervention phase:** Remind all pharmacists and geriatricians participating in the CHARMER trial that the requirement to attend weekly briefings starts this week.  In your communications, also remind them that they may wish to review the benchmarking reports during their briefings. | **Have all participating pharmacists and geriatricians been reminded of the requirement to attend weekly briefings, starting this week?**  Yes  No  **How did you communicate this requirement to the pharmacists and geriatricians?**  Free text |
| Confirm whether the first weekly briefing(s) has taken place as agreed. | **Has the first weekly briefing(s) taken place?**  Yes  No  **Were there any issues or challenges with conducting the weekly briefing? If yes, please describe below.**  Free text |

| **Reporting information (to be submitted by no later than 9 August 2024)** | |
| --- | --- |
| Week 1 briefing(s) | **What was the duration of the weekly briefing(s)?**  Text boxes as time/minutes  Three rows plus option to add more if needed (i.e., as they may have had 3 or more briefings to report on)  ‘No briefings’ option. |
| Is there anything you would like to note about the implementation of the weekly briefings? For example, please note anything you found either particularly challenging or straightforward. | Free text box |

**Resources** [**hyperlinks**]

- Pre-prepared email template to geriatricians and pharmacists
- Deprescribing guide sheet for pharmacists

# 4. Workshop for pharmacists

**Overview of component**

This intervention component comprises a one-hour workshop for pharmacists in which a video with embedded questions provided by the CHARMER team will be shown and discussed. The workshop includes activities to encourage pharmacists to consider the pros and cons of proactive deprescribing and patient case studies to make the benefits of deprescribing, and the harms of failing to deprescribe, more memorable. All pharmacists participating in CHARMER must attend the workshop, which can be delivered online or face-to-face.

**When this needs to happen**

The workshop needs to take place between **1-12 July 2024.** We recommend you begin organising the workshop as soon as possible during the implementation phase.

The workshop **must** be recorded – refer to our guidance [**hyperlink**] and please arrange how you will do this with the CHARMER team or Health Innovation East when you get to this stage.

**What you need to do**

| **Implementation phase (1 May 2024 – 31 July 2024)** | |
| --- | --- |
| Organise a facilitator to run the workshop.  Note: The facilitator must be a Senior Pharmacist or a Geriatrician. Make sure to send the facilitator the following resources to deliver the workshop:   - Facilitator/Project Manager workshop guide [**hyperlink**] - Pre-recorded pharmacist workshop video [**hyperlink**] | **Who will facilitate the workshop?**  Senior Pharmacist  Geriatrician  **Have you sent the facilitator the resources (facilitator workshop guide and pre-recorded video) to deliver the workshop?**  Yes  No  **Does the facilitator have any questions at this stage about their role in facilitating the workshop? If yes, please detail below.**  Free text |
| Identify and invite participants to the workshop. We recommend that at least **three** pharmacists attend the workshop to support discussion.  **Eligible workshop participants to invite:**   - **Mandatory:** All ward-based pharmacists participating in the CHARMER trial - **Optional:** Non-CHARMER ward-based pharmacists covering Older People’s Medicine wards, other pharmacists working for your hospital and nurses.   Geriatricians (other than if facilitating) and medical staff are NOT permitted to attend this workshop. | **Have you invited all pharmacists participating in the CHARMER trial to the workshop?**  Yes  No |
| Agree a suitable date for the workshop.  Note: You must run the workshop **between 1-12 July 2024.** | **What date will the workshop take place?**  Date/calendar  **What time is the workshop scheduled?**  Time |
| If the workshop will be face-to-face/hybrid, book a suitable room.  Note re technology requirements: If running the workshop face-to-face/hybrid, the room will need to have a large computer screen/projector for the facilitator to play the workshop video. | **How will you run the workshop?**  Face-to-face  Online  Hybrid  **Have you booked a suitable room for the workshop?**  Yes  No  Not applicable (online) |
| If one participating pharmacist cannot attend the organised workshop for any reason, organise a one-to-one meeting between the Principal Investigator and that team member to run through the content of the workshop, including the activities.  If two or more participating pharmacists cannot attend the organised workshop, we recommend you either:   - Reschedule the workshop - OR re-run the workshop a second time with the participating pharmacists and any other team members who could not attend, e.g., non-CHARMER ward-based pharmacists covering Older People’s Medicine wards, other pharmacists working for your hospital and nurses.   Speak to the CHARMER team/Health Innovation East if anything is unclear. | **Please detail here any plans if you need to reschedule the workshop, arrange a one-to-one meeting or re-run the workshop a second time:**  Rescheduled workshop details:  One-to-one meeting details:  Second workshop details: |
| The pharmacist workshop must be audio-visually recorded so that we can review attendee engagement with the content. The recording needs to capture ***the majority of the workshop attendees.***  Look over the recording guidance and discuss this with a CHARMER/HIE team member to ensure you have a plan in place of how you will record attendees and how you will send the recording to CHARMER for review. | **Have you discussed your plans for recording the workshop with Health Innovation East/the CHARMER team?**  Yes  No  **What are your plans for recording the workshop?**  Free text |
| Familiarise yourself with the reporting information (see below) you need to capture and submit for this intervention component. | **Are you clear on what reporting information must be collected and submitted for this component?**  Yes  No |

| **Reporting information (to be submitted after the workshop and by no later than 9 August 2024)** | |
| --- | --- |
| Date of pharmacist workshop: | Calendar box |
| How many hours did you (project manager) spend organising the pharmacist workshop? | Free text box |
| How many hours did the facilitator spend preparing to deliver the workshop? | Free text box |
| Names of participating pharmacists who attended the workshop: | Free text box |
| Did all participating pharmacists attend the workshop? | Yes  No (free text box to explain) |
| Please upload the attendance register here (please provide roles only): | Upload |
| How long did the workshop(s) last? List each duration if the workshop ran more than once. | Free text box |
| To the best of your knowledge, was the workshop recorded? | Yes  No |
| Is there anything you would like to note about the implementation of the pharmacist workshop? For example, please note anything you found either particularly challenging or straightforward. | Free text box |

**Resources** [**hyperlinks**]

- Facilitator/Project Manager pharmacist workshop guide
- Pre-recorded pharmacist workshop video
- Attendance register
- Guidance on how to record workshop

# 5. Workshop for geriatricians

**Overview of component**

This intervention component comprises a one-hour workshop for geriatricians in which a video file with embedded questions provided by the CHARMER team will be shown and discussed. The purpose of this intervention component is to address geriatricians’ misconception that patients and carers are resistant to proactive deprescribing by drawing attention to peers who are engaging in successful deprescribing consultations. It also addresses geriatricians’ concerns that primary care colleagues may not welcome deprescribing undertaken in hospital.

All geriatricians participating in CHARMER must attend the workshop. This workshop can be delivered online or face-to-face and can be embedded into an existing slot (e.g., educational meeting).

**When this needs to happen**

The workshop needs to take place between **1-12 July 2024.** We recommend you begin organising the workshop as soon as possible during the implementation phase.

The workshop **must** be recorded – refer to our guidance [**hyperlink**] and please arrange how you will do this with the CHARMER team or Health Innovation East when you get to this stage.

**What you need to do**

| **Implementation phase (1 May 2024 – 31 July 2024)** | |
| --- | --- |
| Organise a facilitator to facilitate the workshop, e.g., a geriatrician.  Note: Make sure you familiarise yourself with the following resources to deliver the workshop:   - Facilitator/Project Manager workshop guide [**hyperlink**] - Pre-recorded geriatrician workshop video [**hyperlink**] | **Who will be facilitating the workshop?**  Principal Investigator  Other (please specify their role, e.g., Geriatrician, Pharmacist):  **Have you sent the facilitator the resources (workshop guide and pre-recorded video) to deliver the workshop?**  Yes  No  **Does the facilitator have any questions at this stage about their role in facilitating the workshop? If yes, please detail below.**  Free text |
| Identify and invite eligible participants to workshop.  **Eligible workshop participants to invite:**   - **Mandatory:** All geriatricians participating in the CHARMER trial - **Optional:** Non-CHARMER geriatricians, junior doctors, pharmacists (including participating CHARMER pharmacists), nurses, allied health professionals | **Have you invited all geriatricians participating in the CHARMER trial to the workshop?**  Yes  No  **If no, please explain why:**  Free text box |
| Agree a suitable date for the workshop.  Note: You must run the workshop between **1-12 July 2024.** | **What date will the workshop take place?**  Date/calendar  **What time is the workshop scheduled?**  Time |
| If the workshop will be face-to-face/hybrid, book a suitable room.  Note re technology requirements: If running the workshop face-to-face/hybrid, the room will need to have a large computer screen/projector for you to play the workshop video. | **How will you run the workshop?**  Face-to-face  Online  Hybrid  **Have you booked a suitable room for the workshop?**  Yes  No  Not applicable (online) |
| If one participating geriatrician cannot attend the organised workshop for any reason, organise a one-to-one meeting between the Principal Investigator and that team member to run through the content of the workshop, including the activities.  If two or more participating geriatricians cannot attend the organised workshop, we recommend you either:   - Reschedule the workshop - OR re-run the workshop a second time with the participating geriatricians and any other team members who could not attend, e.g., non-CHARMER geriatricians, junior doctors, pharmacists (including participating CHARMER pharmacists), nurses, allied health professionals.   Speak to the CHARMER team/Health Innovation East if anything is unclear. | **Please detail here any plans if you need to reschedule the workshop, arrange a one-to-one meeting or re-run the workshop a second time:**  Rescheduled workshop details:  One-to-one meeting details:  Second workshop details: |
| The geriatrician workshop must be audio-visually recorded so that we can review attendee engagement with the content. The recording needs to capture ***the majority of the workshop attendees.***  Look over the recording guidance and discuss this with a CHARMER/HIE team member to ensure you have a plan in place of how you will record attendees and how you will send the recording to CHARMER for review. | **Have you discussed your plans for recording the workshop with Health Innovation East/the CHARMER team?**  Yes  No  **What are you plans for recording the workshop?**  Free text |
| Familiarise yourself with the reporting information (see below) you need to capture and submit for this intervention component. | **Are you clear on what reporting information must be collected and submitted for this component?**  Yes  No |

| **Reporting information (to be submitted after the workshop and by no later than 9 August 2024)** | |
| --- | --- |
| Date of geriatrician workshop: | Calendar box |
| How many hours did you (project manager) spend organising the geriatrician workshop? | Free text box |
| How many hours did the facilitator spend preparing to deliver the workshop? | Free text box |
| Names of participating geriatricians who attended the workshop: | Free text box |
| Names of participating pharmacists who attended the workshop: | Free text box |
| Did all participating geriatricians attend the workshop? | Yes  No (free text box to explain) |
| Please upload the attendance register here (please provide roles only): | Upload |
| How long did the workshop(s) last? List each duration if the workshop ran more than once. | Free text box |
| To the best of your knowledge, was the workshop recorded? | Yes  No |
| Is there anything you would like to note about the implementation of the geriatrician workshop? For example, please note anything you found either particularly challenging or straightforward. | Free text box |

**Resources** [**hyperlinks**]

- Facilitator/Project Manager geriatrician workshop guide
- Pre-recorded geriatrician workshop video
- Attendance register
- Guidance on how to record workshop
