# Supplementary material for: Implementation of a hospital deprescribing behaviour change intervention, the CompreHensive geriAtRician-led MEdication Review (CHARMER) trial: a process evaluation protocol
Source: BMJ Open. 2026 Jun 2;16(6):e111152. doi: 10.1136/bmjopen-2025-111152 (PMC13239467; doi:10.1136/bmjopen-2025-111152)
Supplement: online supplemental file 2 [file bmjopen-16-6-s002.docx]

**CHARMER Site profile questionnaire**

Site Name:

1. IT Infrastructure

What medical records system do you use on the ward(s) taking part in the CHARMER trial? – paper, electronic (please provide name of system), hybrid?

What eprescribing system is used on the ward(s) taking part in the CHARMER trial?

1. Medicines optimisation

Has the site taken part in the last 2 years in any medicines related initiatives that might impact on proactive deprescribing practice? For example, new strategies for medicines reconciliation; patient safety initiatives, CQUINs

If yes, please provide details (including dates and scope)

1. Staff resources at site

Please enter the number of staff members in each of the roles listed below and the total full time equivalent

| Staff Group | Number | FTE |
| --- | --- | --- |
| **Pharmacists** (with ward time on older people’s medicine wards) |  |  |
| **Geriatricians** (with ward time on older people’s medicine wards) (This includes consultants and medical doctors in speciality peoples medicine wards) |  |  |

**CHARMER - PI bi-monthly questionnaires (Steps 1-4)**

We'd appreciate your time in filling out this quick survey to alert us as to whether there have been any key changes relevant to the CHARMER trial at your hospital in the last eight weeks.

1.**Please let us know which site you are reporting from:**

Enter your answer

2.**In the last eight weeks, have there been any changes to the participating geriatricians and pharmacists**(e.g., has a participating clinician taken leave, changed employer, moved ward, reduced their FTE, or decided to withdraw from the study)? Please refer to REDCap to see a list of clinicians participating, and check whether this needs updating.

Yes

No

3.**If yes,** please describe what has changed:
Enter your answer

4.Please confirm that any new geriatricians and pharmacists have been added to REDCap.

Yes

No

5.You can update REDCap here: <https://norwichcrtu.uea.ac.uk/redcap/> Please detail below if you have any issues accessing or updating REDCap.

Enter your answer

6.Please confirm whether the new geriatricians and pharmacists have received the intervention.

Yes

No

7.All new geriatricians and pharmacists need to receive the CHARMER intervention. Please contact the study team at [charmer.study@leicester.ac.uk](mailto:charmer.study@leicester.ac.uk) if you are unclear what you need to do or detail below:

Enter your answer

8.**Please can you confirm the number of wards taking part in the trial and the number of beds on these wards. (eg. if there are 3 wards with a total of 80 beds, enter "3 wards 80 beds")**

Enter your answer

9.**In the last eight weeks**, has there been **any change** to either the number of beds on the study ward(s), or the structure of the study ward(s)? **This includes** any changes to your e-prescribing system, or any medicine optimisation/deprescribing activities at the site other than CHARMER.

Yes

No

10.**If yes,** please describe what has changed:

Enter your answer

11.**Have you started your "Active Intervention" phase?** This is the period following Implementation of the intervention. (Step 1 from August 2024, Step 2 from November 2024, Step 3 from February 2025, and Step 4 from May 2025)

Yes

No

12.Are briefings happening at your hospital?

Yes

No

13. **For the last eight weeks, please estimate how many weekly briefings have taken place on the study ward(s), and their average duration (you may need to contact a participating geriatrician to check this. For example, if there have been 5 briefings with an average duration of 10mins, please enter "5x10mins")**

Enter your answer

14. **Over the last eight weeks, have you had to re-run any of the intervention components (eg. workshops for geriatricians or pharmacists, for rotational staff - how many workshops have you rerun?):**

Enter your answer

15.**If there is anything else you would like to share, please do so here:**

Enter your answer

**CHARMER - PI Updates Step 5 Survey**

We'd appreciate your time in filling out this quick survey to alert us as to whether there have been any key changes relevant to the CHARMER trial at your hospital in the last eight weeks.

1.**Please let us know which site you are reporting from:**

Enter your answer

2.**In the last eight weeks, have there been any changes to geriatricians on your trial ward**(e.g., has the total FTE for geriatricians changed on the ward?)

Yes

No

3.**If yes,** please describe what has changed:

Enter your answer

4.**Please can you confirm the number of wards taking part in the trial and the number of beds on these wards. (eg. if there are 3 wards with a total of 80 beds, enter "3 wards 80 beds")**.

Enter your answer

5.**In the last eight weeks**, has there been **any change** to either the number of beds on the study ward(s), or the structure of the study ward(s)? **This includes** any changes to your e-prescribing system, or any medicine optimisation/deprescribing activities at the site.

Yes

No

6.**If yes,** please describe what has changed:

Enter your answer

7.**If there is anything else you would like to share, please do so here:**

Enter your answer

**Questionnaire about barriers and enablers to implementing the CHARMER intervention (Project manager completion)**

For completion by project manager supporting implementation of the CHARMER intervention.

Thank you for supporting implementation of the CHARMER intervention at your hospital.

**We would like to ask you about your thoughts on anything that helped or hindered implementation and impact of CHARMER at your hospital.**

**The survey should take approximately 10 minutes to complete and all detail you can provide is useful.** Anything you tell us will only be seen by members of the CHARMER research team; we will not share the information with your hospital.

Please tell us about any challenges experienced when implementing CHARMER at your hospital?

Please tell us about anything that helped when implementing CHARMER at your hospital?

Please tell us any recommendations you have for facilitating future roll-out of CHARMER (if CHARMER is shown to be effective and cost-effective).

Is there anything else you would like to tell us that is not covered above?

**Thank you for taking the time to complete this survey.**

**Practitioner Demographics**

Participant ID:

**Site:**

**Collect:**

Age:

Gender :

Ethnicity: NHS 16+ 1 categories

Occupational Group:

Job Role:

Grade or Banding (e.g. Consultant or Band 7):

Years at Current Grade/Banding:

Previous Training or education relevant to deprescribing (provide date and scope):

**Practitioner deprescribing questionnaire**

Thank you for taking part in the CHARMER study. As part of your participation, we would like to hear about your experience of deprescribing medicines for older people in your hospital. This questionnaire should take approximately 10 minutes to complete.

Please read each question and respond by ticking the appropriate box according to: always, often, sometimes, rarely, never.

| **Practitioner deprescribing** | | | | | |
| --- | --- | --- | --- | --- | --- |
| **Identifying a patient for potential stop of a medicine** | | | | | |
|  | always | often | sometimes | rarely | never |
| 1. How often do you review medicines for continued appropriateness for every patient under your care? |  |  |  |  |  |
| 1. When starting a medication, how often do you explain to the patient/relative that it will be reviewed regularly and stopped if no longer suitable? |  |  |  |  |  |
| **Evaluating a patient for potential stop of a medicine** | | | | | |
|  | always | often | sometimes | rarely | never |
| 1. How often do you ask the patient/relative to disclose all medications they are taking? |  |  |  |  |  |
| 1. How often do you evaluate if the patient/relative is taking their medication as prescribed? |  |  |  |  |  |
| 1. How often do you ask the patient/relative about their thoughts and experiences of taking their medication? |  |  |  |  |  |
| 1. How often do you ask if the medication is meeting the patient's goals and priorities? |  |  |  |  |  |
| 1. How often do you consider the patient's life expectancy when evaluating a patient for potential stop of a medicine? |  |  |  |  |  |
| 1. How often do you consider non-pharmacological options when evaluating a patient for potential stop of a medicine? |  |  |  |  |  |
| 1. How often do you consider the likelihood of benefit and harm from the prescribed medicine(s)? |  |  |  |  |  |
| 1. How often do you consider the likelihood of benefit and harm from stopping the prescribed medicine(s)? |  |  |  |  |  |
| 1. How often do you provide the patient/relative with the information gathered about their factors that are relevant to stopping their medicine? |  |  |  |  |  |
| **Stopping a medicine** | | | | | |
|  | always | often | sometimes | rarely | never |
| 1. How often do you invite the patient/relative to decide if they would like to stop a medicine? |  |  |  |  |  |
| 1. How often do you establish whether immediate withdrawal or a tapered or weaning approach is needed when stopping a medicine? |  |  |  |  |  |
| 1. How often do you record a plan for stopping the medicine in the patient's notes or record? |  |  |  |  |  |
| **After a medicine has been stopped** | | | | | |
| 1. How often do you arrange follow up appointments after a medicine has been stopped? |  |  |  |  |  |
| 1. How often do you monitor the patient for adverse drug withdrawal effects after a medicine has been stopped? |  |  |  |  |  |
| 1. How often do you monitor the patient's quality of life once a medicine has been stopped? |  |  |  |  |  |
| 1. How often do you feel that patients/relatives are successfully involved in the above process of stopping medicines before harm occurs? |  |  |  |  |  |
| **Time required (minutes)** | <2 | 2 to 5 | 6 to 10 | 11 to 15 | >15 |
| 1. How long on average are your discussions with a patient about possibly reducing or stopping a medication? |  |  |  |  |  |

**Thank you for taking the time to answer these questions.**

**Deprescribing Behaviour Change Mechanism of Action Questionnaire**

Participant ID:

Baseline/Follow-up (delete as appropriate)

1. I have the knowledge I need to enable me to make the right deprescribing decision/recommendations for my patients’ medication.

| Strongly disagree | Disagree | Neither agree nor disagree | Agree | Strongly agree |
| --- | --- | --- | --- | --- |
|  |  |  |  |  |

1. Proactive deprescribing has more advantages than disadvantages

| Strongly disagree | Disagree | Neither agree nor disagree | Agree | Strongly agree |
| --- | --- | --- | --- | --- |
|  |  |  |  |  |

1. I feel confident that I could make the right deprescribing decision/recommendations for my patients’ medication.

| Strongly disagree | Disagree | Neither agree nor disagree | Agree | Strongly agree |
| --- | --- | --- | --- | --- |
|  |  |  |  |  |

1. Proactive deprescribing is a clinical practice that is accepted by patients and carers

| Strongly disagree | Disagree | Neither agree nor disagree | Agree | Strongly agree |
| --- | --- | --- | --- | --- |
|  |  |  |  |  |

1. I have the skills I need to enable me to have effective consultations with patients and/or their carers about proactive deprescribing of their medication.

| Strongly disagree | Disagree | Neither agree nor disagree | Agree | Strongly agree |
| --- | --- | --- | --- | --- |
|  |  |  |  |  |

1. If I did proactively deprescribe or recommend proactive deprescribing I would worry about it after.

| Strongly disagree | Disagree | Neither agree nor disagree | Agree | Strongly agree |
| --- | --- | --- | --- | --- |
|  |  |  |  |  |

1. Making proactive deprescribing decisions and/or recommendations is consistent with what is expected of my role.

| Strongly disagree | Disagree | Neither agree nor disagree | Agree | Strongly agree |
| --- | --- | --- | --- | --- |
|  |  |  |  |  |

1. I can actively support deprescribing within my regular workload

| Strongly disagree | Disagree | Neither agree nor disagree | Agree | Strongly agree |
| --- | --- | --- | --- | --- |
|  |  |  |  |  |

1. My trust has a clear policy for deprescribing

| Strongly disagree | Disagree | Neither agree nor disagree | Agree | Strongly agree |
| --- | --- | --- | --- | --- |
|  |  |  |  |  |

1. My department has a clear policy for deprescribing

| Strongly disagree | Disagree | Neither agree nor disagree | Agree | Strongly agree |
| --- | --- | --- | --- | --- |
|  |  |  |  |  |

1. I feel that deprescribing efforts in my trust are noticed

| Strongly disagree | Disagree | Neither agree nor disagree | Agree | Strongly agree |
| --- | --- | --- | --- | --- |
|  |  |  |  |  |
