# Supplementary material for: Implementation of a hospital deprescribing behaviour change intervention, the CompreHensive geriAtRician-led MEdication Review (CHARMER) trial: a process evaluation protocol
Source: BMJ Open. 2026 Jun 2;16(6):e111152. doi: 10.1136/bmjopen-2025-111152 (PMC13239467; doi:10.1136/bmjopen-2025-111152)
Supplement: online supplemental file 3 [file bmjopen-16-6-s003.docx]

**The process of having your medicine(s) stopped: How satisfied are you?**

Participant ID:

|  |  | / |  |  | / |  |  |  |  |
| --- | --- | --- | --- | --- | --- | --- | --- | --- | --- |

Date completed:

**Instructions to provide to participant:**

During your stay in hospital, you kindly agreed to take part in the CHARMER study. The study is testing a support package to help doctors and pharmacists stop medicines that patients no longer need. We are asking you to complete this questionnaire about your experience of the process of having one or more of your medicine(s) stopped.

This questionnaire has 11 questions and will take less than 5 minutes to answer.

There are no right or wrong answers so please give your honest views.

Please complete this questionnaire on your own or with help from a relative, friend or a research nurse.

Read each question and then pick from the answers to say how satisfied or dissatisfied you are with each part of the process of having your medicine(s) stopped.

If a question does not apply to you, for example because that part of the process did not happen, please pick the answer 'Not applicable'.

Your answers will be kept strictly confidential and will only be seen by the Research Nurse and members of the CHARMER research team.

1.

|  | Myself | A healthcare professional | Someone else | Not sure |
| --- | --- | --- | --- | --- |
| Who first suggested stopping the medication? | ☐ | ☐ | ☐ | ☐ |

2.

|  | Very dissatisfied | Somewhat dissatisfied | Neither satisfied nor dissatisfied | Somewhat satisfied | Very satisfied | Not applicable |
| --- | --- | --- | --- | --- | --- | --- |
| How satisfied are you that the healthcare professional was aware of all the medicines that you were taking? | ☐ | ☐ | ☐ | ☐ | ☐ | ☐ |

3.

|  | Very dissatisfied | Somewhat dissatisfied | Neither satisfied nor dissatisfied | Somewhat satisfied | Very satisfied | Not applicable |
| --- | --- | --- | --- | --- | --- | --- |
| How satisfied are you that the healthcare professional encouraged you to share any positive or negative aspects of taking each of your medicines? | ☐ | ☐ | ☐ | ☐ | ☐ | ☐ |

4.

|  | Very dissatisfied | Somewhat dissatisfied | Neither satisfied nor dissatisfied | Somewhat satisfied | Very satisfied | Not applicable |
| --- | --- | --- | --- | --- | --- | --- |
| How satisfied are you that non-medicine alternatives to treating your health condition(s) were considered? | ☐ | ☐ | ☐ | ☐ | ☐ | ☐ |

5.

|  | Very dissatisfied | Somewhat dissatisfied | Neither satisfied nor dissatisfied | Somewhat satisfied | Very satisfied | Not applicable |
| --- | --- | --- | --- | --- | --- | --- |
| How satisfied are you that the healthcare professional thought about the benefits of continuing to take your medicine(s) with all the possible side effects in mind? | ☐ | ☐ | ☐ | ☐ | ☐ | ☐ |

6.

|  | Very dissatisfied | Somewhat dissatisfied | Neither satisfied nor dissatisfied | Somewhat satisfied | Very satisfied | Not applicable |
| --- | --- | --- | --- | --- | --- | --- |
| How satisfied are you that the possible benefits and harm from stopping the medicine(s) were considered? | ☐ | ☐ | ☐ | ☐ | ☐ | ☐ |

7.

|  | Very dissatisfied | Somewhat dissatisfied | Neither satisfied nor dissatisfied | Somewhat satisfied | Very satisfied | Not applicable |
| --- | --- | --- | --- | --- | --- | --- |
| How satisfied are you with how the healthcare professional’s reasons for stopping your medicine(s) were explained? | ☐ | ☐ | ☐ | ☐ | ☐ | ☐ |

8.

|  | Very dissatisfied | Somewhat dissatisfied | Neither satisfied nor dissatisfied | Somewhat satisfied | Very satisfied | Not applicable |
| --- | --- | --- | --- | --- | --- | --- |
| How satisfied are you that you were given enough opportunity to decide whether you wanted to stop your medicine(s)? | ☐ | ☐ | ☐ | ☐ | ☐ | ☐ |

9.

|  | Very dissatisfied | Somewhat dissatisfied | Neither satisfied nor dissatisfied | Somewhat satisfied | Very satisfied | Not applicable |
| --- | --- | --- | --- | --- | --- | --- |
| How satisfied are you that the healthcare professional made a plan for stopping one or more of your medicines? | ☐ | ☐ | ☐ | ☐ | ☐ | ☐ |

10.

|  | Very dissatisfied | Somewhat dissatisfied | Neither satisfied nor dissatisfied | Somewhat satisfied | Very satisfied |
| --- | --- | --- | --- | --- | --- |
| **Overall**, how satisfied are you with the process of having a medicine stopped by a healthcare professional? | ☐ | ☐ | ☐ | ☐ | ☐ |

11.

Use the space below to provide any comments about how your medicine(s) were stopped.

|  |
| --- |

**Thank you for taking the time to answer these questions, your feedback is valuable to us.**

Please hand the completed questionnaire back to the person who gave it to you.

**The process of your relative’s or friend’s medicines being stopped: How satisfied are you?**

Participant ID:

|  |  | / |  |  | / |  |  |  |  |
| --- | --- | --- | --- | --- | --- | --- | --- | --- | --- |

Date completed:

**Instructions to provide to participant:**

During your friend/relative’s stay in hospital, you kindly agreed to them taking part in the CHARMER study. The study is testing a support package to help doctors and pharmacists stop medicines that patients no longer need. We are asking you to complete this questionnaire about your experience of the process of your relative’s or friend’s medicine(s) being stopped by a healthcare professional.

This questionnaire has 11 questions and will take less than 5 minutes to answer.

There are no right or wrong answers so please give your honest views.

Read each question and then pick from the answers to say how satisfied or dissatisfied you are with each part of the process of your relative’s or friend’s medicine(s) being stopped.

If a question does not apply, for example because that part of the process did not happen or you were not involved in that part, please pick the answer 'Not applicable'.

Your answers will be kept strictly confidential and will only be seen by the Research Nurse and members of the CHARMER research team.

1.

|  | Myself | A healthcare professional | Someone else | Not sure |
| --- | --- | --- | --- | --- |
| Who first suggested stopping the medication? | ☐ | ☐ | ☐ | ☐ |

2.

|  | Very dissatisfied | Somewhat dissatisfied | Neither satisfied nor dissatisfied | Somewhat satisfied | Very satisfied | Not applicable |
| --- | --- | --- | --- | --- | --- | --- |
| How satisfied are you that the healthcare professional was aware of all the medicines that your relative or friend was taking? | ☐ | ☐ | ☐ | ☐ | ☐ | ☐ |

3.

|  | Very dissatisfied | Somewhat dissatisfied | Neither satisfied nor dissatisfied | Somewhat satisfied | Very satisfied | Not applicable |
| --- | --- | --- | --- | --- | --- | --- |
| How satisfied are you that the healthcare professional encouraged your relative or friend to share any positive or negative aspects of taking each of their medicines? | ☐ | ☐ | ☐ | ☐ | ☐ | ☐ |

4.

|  | Very dissatisfied | Somewhat dissatisfied | Neither satisfied nor dissatisfied | Somewhat satisfied | Very satisfied | Not applicable |
| --- | --- | --- | --- | --- | --- | --- |
| How satisfied are you that non-medicine alternatives to treating your relative’s or friend’s health condition(s) were considered? | ☐ | ☐ | ☐ | ☐ | ☐ | ☐ |

5.

|  | Very dissatisfied | Somewhat dissatisfied | Neither satisfied nor dissatisfied | Somewhat satisfied | Very satisfied | Not applicable |
| --- | --- | --- | --- | --- | --- | --- |
| How satisfied are you that the healthcare professional thought about the benefits of your relative or friend continuing to take their medicine(s) with all the possible side effects in mind? | ☐ | ☐ | ☐ | ☐ | ☐ | ☐ |

6.

|  | Very dissatisfied | Somewhat dissatisfied | Neither satisfied nor dissatisfied | Somewhat satisfied | Very satisfied | Not applicable |
| --- | --- | --- | --- | --- | --- | --- |
| How satisfied are you that the possible benefits and harm from your relative or friend stopping their medicine(s) were considered? | ☐ | ☐ | ☐ | ☐ | ☐ | ☐ |

7.

|  | Very dissatisfied | Somewhat dissatisfied | Neither satisfied nor dissatisfied | Somewhat satisfied | Very satisfied | Not applicable |
| --- | --- | --- | --- | --- | --- | --- |
| How satisfied are you with how the healthcare professional’s reasons for stopping your relative’s or friend’s medicine(s) were explained? | ☐ | ☐ | ☐ | ☐ | ☐ | ☐ |

8.

|  | Very dissatisfied | Somewhat dissatisfied | Neither satisfied nor dissatisfied | Somewhat satisfied | Very satisfied | Not applicable |
| --- | --- | --- | --- | --- | --- | --- |
| How satisfied are you that enough opportunity was given to decide whether to stop your relative’s or friend’s medicine(s)? | ☐ | ☐ | ☐ | ☐ | ☐ | ☐ |

9.

|  | Very dissatisfied | Somewhat dissatisfied | Neither satisfied nor dissatisfied | Somewhat satisfied | Very satisfied | Not applicable |
| --- | --- | --- | --- | --- | --- | --- |
| How satisfied are you that the healthcare professional made a plan for stopping one or more of your relative’s or friend’s medicines? | ☐ | ☐ | ☐ | ☐ | ☐ | ☐ |

10.

|  | Very dissatisfied | Somewhat dissatisfied | Neither satisfied nor dissatisfied | Somewhat satisfied | Very satisfied |
| --- | --- | --- | --- | --- | --- |
| **Overall**, how satisfied are you with the process of your relative’s or friend’s medicines being stopped by a healthcare professional? | ☐ | ☐ | ☐ | ☐ | ☐ |

11.

Use the space below to provide any comments about how your relative’s or friend’s medicine(s) were stopped.

|  |
| --- |

**Thank you for taking the time to answer these questions, your feedback is valuable to us.**

Please hand the completed questionnaire back to the person who gave it to you.
