# Supplementary material for: Implementation of a hospital deprescribing behaviour change intervention, the CompreHensive geriAtRician-led MEdication Review (CHARMER) trial: a process evaluation protocol
Source: BMJ Open. 2026 Jun 2;16(6):e111152. doi: 10.1136/bmjopen-2025-111152 (PMC13239467; doi:10.1136/bmjopen-2025-111152)
Supplement: online supplemental file 4 [file bmjopen-16-6-s004.docx]

# TOPIC GUIDES, 06-03-2025

# Primary care practitioners with a prescribing role [30 minutes]

Introduction:

I am a researcher working on the CHARMER trial. The CHARMER trial is testing an intervention to support geriatricians and pharmacists to proactively deprescribe unnecessary or harmful medicines for older people in hospital.

I am keen to hear your thoughts on how the CHARMER intervention from your perspective in primary care– both positive and negative aspects. There are no right or wrong answers. The information you provide will help us to understand what worked well and what could be improved.

We will not ask you to disclose patient information during the interview, the questions will focus more on your experience of patients, who were recruited in the CHARMER trial, after their hospital discharge. We would like to hear your thoughts about the impact of CHARMER on primary care. We will also about your experience as a primary care clinician more generally when patients have had a medicine stopped in hospital.

Today I will be recording our discussion but only myself, the researchers working on the study and the person who transcribes the interview will hear what we say. When the interview is transcribed, we remove any identifiable information such as your name so that we have an anonymised record of what we talked about. After the interview is transcribed we will securely destroy the recording of the interview.

If we use any quotes from you in our reports, you will not be able to be identified.

You can also stop the interview at any time and without giving a reason.

Do you have any questions for me before we start?

**Can you tell me a bit about yourself and your role?**

**How do you feel about medicines being stopped in hospital?**

**With the patients who were involved in CHARMER, what was your experience after the patient was discharged from hospital?**

*Prompts:*

- *Was it clear why the medicine was stopped in hospital? What information were you provided with (discharge summary etc)?*
- *Were deprescribing decisions made in hospital maintained/implemented or changed?*
- *How do you think the patient’s experience was? Did they have questions about their medication after they left hospital?*
- *If we had not had this interview, would you have been aware that some of your patients had had a medicine(s) stopped in hospital?*

**Please describe to us, the primary care process of responding to a patient’s medicine(s) being stopped in hospital**

*Prompts:*

- *What happens/how do you know a patient has had a medicine(s) stopped in hospital? (do you receive an alert, or are you directed to review a discharge summary etc?)*
- *In general. what does the discharge summary contain? Does it contain enough detail about why the medicines was stopped?*
- *Do you ever need to phone the hospital to find out more information about why the medicine was stopped?*
- *How often are you managing a patient who has had a medicine stopped in hospital?*

**As a primary care clinician, what are your thoughts on deprescribing happening in hospital?**

*Prompts:*

- *What are your thoughts on the best place and time to stop a patient’s medicine?*
- *Do you ever feel concerned about a patient’s medicine being stopped in hospital?*
- *Some geriatricians and pharmacists have told us that they sometimes worry about potential conflict with primary care if they stop a patient’s medicine, or they worry that stopping a medicine may be more appropriate to do in primary care than in hospital. What do you think about this?*

**Can you foresee any impact (positive or negative) of CHARMER on primary care (increased number of patients having a medication(s) stopped in hospital)?**

*Prompts:*

- *any challenges for you / primary care as a result of CHARMER?*
- *anything that would need to change to make the process better?*

**Is there anything we haven’t covered today that you would like to discuss?**

# Project manager (Implementation) – after implementation [60 minutes]

Introduction: I am a researcher working on the CHARMER trial.

Today I’d like to ask you about your experience of being involved in the implementation/delivery of the CHARMER study. I am keen to hear your thoughts on how the study went from your perspective – both positive and negative aspects. There are no right or wrong answers. The information you provide will help us to understand what worked well and what could be improved.

Today I will be recording our discussion but only myself, the researchers working on the study and the person who transcribes the interview will hear what we say. When the interview is transcribed, we remove any identifiable information such as your name so that we have an anonymised record of what we talked about. After the interview is transcribed, we will securely destroy the recording of the interview.

If we use any quotes from you in our reports, you will not be able to be identified.

You can also stop the interview at any time and without giving a reason.

Do you have any questions for me before we start?

**Can you tell me a bit about yourself and your role?**

*Have you been involved in research previously? If yes, what type of study and what was your involvement? Did that previous experience help you with working on CHARMER?*

**If not research focussed* Were you already familiar with how the R&D team at your site supports clinical research? *If no, do you understand more about how*

*Had any of the PIs at your site been involved in research before? Were any acting in this capacity for the first time?*

**Can you tell me about your experience of being involved in the CHARMER study?**

*Prompts:*

*How did you find the study?*

*What worked well? What worked less well?*

*Have you been involved in research previously? If yes, what type of study and what was your involvement? Did that previous experience help you with working on CHARMER?*

**If not research focussed* Were you already familiar with how the R&D team at your site supports clinical research? *If no, do you understand more about how*

*Had any of the PIs at your site been involved in research before? Were any acting in this capacity for the first time?*

**Can you tell me about the implementation of the CHARMER intervention?**

*Prompts:*

*How were each of the intervention components implemented?*

*Were any of the intervention components changed when implemented? If yes, why did this happen?*
*Were there challenges with organising implementation? How were these overcome?*

*What aspects helped with implementation?*

*How did the pharmacists and geriatricians participate/engage with each of the (relevant) intervention components? Did they engage more with particular components?*

*Have geriatricians and pharmacists engaged with the benchmarking and briefings?*

**What do you think of the CHARMER intervention?**

*Prompts:*

*What did you think of each component?*

*If pharmacist/medical: how do you feel CHARMER impacts on/supports proactive deprescribing?*

*Now that you have implemented CHARMER, how would you describe it to other people?*

**Can you tell me about the information you received that supported implementation of the CHARMER intervention?**

*Prompts:*

*What aspects did you find useful?*

*What aspects do you think could be improved?*

*What was your experience of using REDCap*? *Any issues with adding data in real time? Any challenges with accessing or using REDCap?*

**Do you feel there were any factors at your site that either helped or hindered the CHARMER intervention?**

*Prompts:* *This could be related to for example, contextual information such as features of the hospital or study ward, people involved, processes etc*

**What impact do you think the CHARMER intervention has had?**

*Prompts:*

*Have you noticed any changes with staff since CHARMER has happened? If yes, what have you noticed?*

*Have you noticed any changes/questions by patients or family because they are aware of CHARMER?*

**If research is not already their full role* Has your experience with CHARMER made you more or less likely to take part in* future opportunities to deliver research (or no difference)?

**Is there anything else that you would like to discuss that we have not covered?**

# Recruited practitioners – geriatricians and pharmacists during intervention phase [60 minutes]

Introduction:

I am a researcher working on the CHARMER trial.

Today I’d like to ask you about your experience of being involved in the CHARMER trial.

I am keen to hear your thoughts on how the study went from your perspective – both positive and negative aspects. There are no right or wrong answers. The information you provide will help us to understand what worked well and what could be improved.

Today I will be recording our discussion but only myself, the researchers working on the study and the person who transcribes the interview will hear what we say. When the interview is transcribed, we remove any identifiable information such as your name so that we have an anonymised record of what we talked about. After the interview is transcribed, we will securely destroy the recording of the interview.

If we use any quotes from you in our reports, you will not be able to be identified.

You can also stop the interview at any time and without providing a reason.

Do you have any questions for me before we start?

**Can you tell be a bit about yourself and your role at the hospital?**

*Prompts:*

*How long have you worked at the hospital?*

**Can you tell me about your experience in the CHARMER trial?**

*Prompts:*

*Why did you decide to take part?
Was it clear why CHARMER was happening at your hospital and what the purpose of CHARMER is?*

*What went well? What was challenging?*

*What types of research have you previously been involved in?*

**What are your thoughts on proactive deprescribing?**

*Is proactive deprescribing a key part of your role?*

*How do you feel CHARMER affects proactive deprescribing?*

*Are there any other things that stop you doing more proactive deprescribing?*

*Where and when do you think it is best to undertake proactive deprescribing?*

**What do you think of the CHARMER intervention?**

*Prompts:*

*Action plan*

*2. Workshop*

*3. Briefings & benchmarking during intervention person*

*What did you think about the different components of the intervention?*

*Do you think all components were useful? Did all components make sense? Do you think any changes are needed?*

**What happened with briefings and benchmarking?**

*Prompts:*

*How did you find the weekly briefings and benchmarking reports? Were these useful? How did you meet each week?*

*How have these happened and have they changed over time?*

*Have you noticed changes in how geriatricians and pharmacists work together? If yes, can you describe the changes?*

**What impact do you think the CHARMER intervention has had?**

*Prompts:*

*How has CHARMER changed your practice and work?*

*How has CHARMER affected how you work with geriatricians/pharmacists (– as appropriate)?*

*How has CHARMER made a difference to how you undertake proactive deprescribing?*

*How has CHARMER changed how you discuss medication changes with your patients or with colleagues?*

*Do you think any impact (that participant notes) is likely to be sustained?*

**Do you feel there were any factors at your site that either helped or hindered the intervention or proactive deprescribing?**

*Prompts: This could be related to for example, contextual information such as features of the hospital or study ward, the people involved, processes, infrastructure/IT etc*

*How have these factors affected CHARMER or proactive deprescribing?*

*Do you think there are barriers to proactive deprescribing?*

*Has your experience with CHARMER made you more or less likely to take part in future opportunities to deliver research (or no difference)?*

**Is there anything else that you would like to discuss that we have not covered?**

# Principal Investigator [60 minutes] – end of trial

Introduction: I am a researcher working on the CHARMER trial.

Today I’d like to ask you about your experience of being involved in the CHARMER trial. I am keen to hear your thoughts on how the study went from your perspective – both positive and negative aspects. There are no right or wrong answers. The information you provide will help us to understand what worked well and what could be improved.

Today I will be recording our discussion but only myself, the researchers working on the study and the person who transcribes the interview will hear what we say. When the interview is transcribed, we remove any identifiable information such as your name so that we have an anonymised record of what we talked about. After the interview is transcribed, we will securely destroy the recording of the interview.

If we use any quotes from you in our reports, you will not be able to be identified.

You can also stop the interview at any time and without giving a reason.

Do you have any questions for me before we start?

**Can you tell me about you and your role at the hospital?**

**Can you tell me about your experience of being involved in the CHARMER study?**

*Prompts:*

- *What went well? What was challenging?*
- *Have you been involved in other research – if yes, what type of study and what was your involvement? Did that previous experience help you with working on CHARMER?*
- *How did you find the trial activities and intervention activities happening alongside each other?*

**How did pharmacists and geriatricians respond to CHARMER?**

*Prompts:*

*Who/how many have received the intervention*

*How many are still in a position to proactively deprescribe on intervention wards?*

*Where geriatricians or pharmacists left, did you replace them? If not, why not? What were the challenges?*

*How did geriatricians and pharmacists respond to being asked to participate in and receive CHARMER?*

*Did you attend any of the implementation events (action plan, workshops)? If yes, how did you feel pharmacists/geriatricians engaged with the components? – What you thought of action plan and workshops etc.*

*What have you noticed over time? (both staff behaviour? Enthusiasm? Deprescribing?)*

**What happened with briefings and benchmarking?**

*Prompts:*

*How have these happened and have they changed over time?*

*Challenges / things that went well with setting it up*

*Have you noticed changes in how geriatricians and pharmacists work together? If yes, can you describe the changes?*

**What impact do you think the CHARMER intervention has had?**

*Prompts:*

*How has CHARMER changed your practice and work?*

*How has CHARMER affected how you work with geriatricians/pharmacists (– as appropriate)?*

*How has CHARMER made a difference to how you undertake proactive deprescribing?*

*How has CHARMER changed how you discuss medication changes with your patients or with colleagues?*

*Do you think any impact (that participant notes) is likely to be sustained?*

*If stopped doing briefings / benchmarking – explore when that happened and why?*

**Do you feel there were any factors at your site that either helped or hindered the CHARMER intervention?**

*Prompts: This could be related to for example, contextual information such as features of the hospital or study ward, people involved, processes, infrastructure/IT etc*

**What are your thoughts on proactive deprescribing?**

*Is proactive deprescribing a key part of your role?*

*How do you feel CHARMER affects proactive deprescribing?*

*Are there any other things that stop you doing more proactive deprescribing?*

***Where and when do you think it is best to undertake proactive deprescribing?***

**Now that you have implemented CHARMER, how would you describe it to other people?**

**If research is not their full role** **Has your experience with CHARMER made you more or less likely to take part in future opportunities to deliver research (or no difference)?**

*Only if fitting* Has it helped you to feel more equipped to deliver research in the future?

**Is there anything else that you would like to discuss that we have not covered?**

# Clinical Research Nurses (Ageing Specialty Research Staff) involved in patient recruitment [30 minutes]

Introduction:

I am a researcher working on the CHARMER trial which is testing an intervention to support geriatricians and pharmacists to proactively deprescribe unnecessary or harmful medicines for older people in hospital.

Today I’d like to ask you about your experience of being involved in the CHARMER study. I am keen to hear your thoughts on how the study went from your perspective – both positive and negative aspects. There are no right or wrong answers. The information you provide will help us to understand what worked well and what could be improved.

Today I will be recording our discussion but only myself, the researchers working on the study and the person who transcribes the interview will hear what we say. When the interview is transcribed, we remove any identifiable information such as your name so that we have an anonymised record of what we talked about. After the interview is transcribed we will securely destroy the recording of the interview.

If we use any quotes from you in our reports, you will not be able to be identified.

You can also stop the interview at any time and without giving a reason.

Do you have any questions for me before we start?

**Can you tell me about your role in the CHARMER study?**

**Can you tell me about your experience of being involved in the CHARMER study?**

*Prompts:*

*How did you find being involved in the study?*

*How did you find communication with the CHARMER team? Is there anything we could have done differently to support you in your role?*

*Were you provided with all the resources you needed for your role?*

**How did you find the process of recruiting patients and consultees into the study?**

*Prompts:*

*What worked well? What was challenging? Could any processes be changed to increase recruitment?*

*What questions, if any, did patients and their families have about the study?*

**Can you tell me about your experiences of collecting data within the CHARMER study?**

*Prompts: Were there data that were easier to collect?*

*Were any data items burdensome to collect?*

*Could we make any changes to data collection processes, if so what?*

*How was your experience of the collection of data from patients and consultees?*

*Some of the data collection was by phone, how did this go?*

**What resources were needed for recruitment and data collection in the CHARMER trial?**

*Prompts: how many staff?, how much resource was given to recruitment and data collection?* **Do you feel there were any factors at your site that either helped or hindered the CHARMER trial?**

*Prompts:* *This could be related to for example, contextual information such as features of the hospital or study ward, people involved, processes, infrastructure/IT etc*

**What impact do you think the CHARMER intervention has had?**

*Prompts:*

*Have you noticed any changes/questions by patients or family because they are aware of CHARMER?*

*Have you noticed any changes with staff since CHARMER has happened?*

**Is there anything else that you would like to discuss that we have not covered?**

# Patients [up to 20 minutes]

***Interview 1:***

Introduction:

I am a researcher working on the CHARMER trial. The CHARMER trial is testing a method to support doctors and pharmacists to review and stop unnecessary or harmful medicines. I’m interviewing you because you were at one of the hospitals that took part in CHARMER and I’d like to hear your thoughts about your experience in hospital.

I am keen to hear your thoughts on how the study went from your perspective – both positive and negative aspects. There are no right or wrong answers. The information you provide will help us to understand what worked well and what could be improved.

Today I will be recording our discussion but only myself, the researchers working on the study and the person who transcribes the interview will hear what we say. When the interview is transcribed, we remove any identifiable information such as your name so that we have an anonymised record of what we talked about. After the interview is transcribed we will securely destroy the recording of the interview.

If you do disclose anything which might identify a risk to yourself or to others, I would have a duty to let someone know, such as your GP, but I would tell you if I thought this were the case.

If we use any quotes from you in our reports, you will not be able to be identified.

You can also stop the interview at any time and without giving a reason.

Do you have any questions for me before we start?

**Can you tell me what led to you going into hospital and how long you stayed in hospital?**

**Can you tell me about what happened whilst you were in hospital?**

**Whilst you were in hospital, did the healthcare staff discuss with you about stopping one or more of your medicines?**

*Prompt: What can you remember about that conversation?*

**Whilst you were in hospital were any of your medicines stopped?**

*Prompt: How have you been getting on with that?*

**Since you left hospital, what has happened?**

**Is there anything we haven’t covered today that you would like to discuss?**

**Would it be OK to call you back in about 3 months' time to talk to you again about any discussions regarding your medication?**

# Patients 2 [up to 20 minutes]

***Interview 2:***

Introduction:

I am a researcher working on the CHARMER trial and we spoke X months ago. The CHARMER trial is testing a method to support doctors and pharmacists to review and stop unnecessary or harmful medicines. I’m interviewing you because you were at one of the hospitals that took part in CHARMER and I am keen to hear your thoughts on what has happened since your last interview with me – both positive and negative aspects. There are no right or wrong answers. The information you provide will help us to understand what worked well and what could be improved.

Today I will be recording our discussion but only myself, the researchers working on the study and the person who transcribes the interview will hear what we say. When the interview is transcribed, we remove any identifiable information such as your name so that we have an anonymised record of what we talked about. After the interview is transcribed we will securely destroy the recording of the interview.

If you do disclose anything which might identify a risk to yourself or to others, I would have a duty to let someone know, such as your GP, but I would tell you if I thought this were the case.

If we use any quotes from you in our reports, you will not be able to be identified.

You can also stop the interview at any time and without giving a reason.

Do you have any questions for me before we start?

So today I am interested in hearing what has happened since we last met. Last time, you had left hospital X weeks before.

**Since we met last for an interview, what has happened?**

**Have you seen your GP or pharmacist since we last spoke?**

***Last time you told me that some of your medicines changed in hospital.* Have any of your medicines been re-started since we last spoke?**

*If ‘yes’ - Who reinstated them?*

*What happened during that conversation?*

*If ‘No’ – Have you discussed your medicine changes with a healthcare professional since leaving hospital?*

**How did you feel about the changes made to your medicines?**

*How have you been getting on since the changes?*

**Part of being in the study has meant that you have phone calls with a nurse, and they ask you about your thoughts and feelings about how your current health impacts your daily life. How did/do you feel about answering these questions?**

*What sort of things did you discuss?*

**Is there anything we haven’t covered today that you would like to discuss?**

# Consultees [up to 20 minutes]

**Interview 1**

Introduction:

I am a researcher working on the CHARMER trial. The CHARMER trial is testing a method to support doctors and pharmacists to review and stop unnecessary or harmful medicines. I’m interviewing you because you were at one of the hospitals that took part in CHARMER and I’d like to hear your thoughts about your relative’s experience in hospital.

I am keen to hear your thoughts on how the study went from your perspective – both positive and negative aspects. There are no right or wrong answers. The information you provide will help us to understand what worked well and what could be improved.

Today I will be recording our discussion but only myself, the researchers working on the study and the person who transcribes the interview will hear what we say. When the interview is transcribed, we remove any identifiable information such as your name so that we have an anonymised record of what we talked about. After the interview is transcribed we will securely destroy the recording of the interview.

If you do disclose anything which might identify a risk to yourself or to others, I would have a duty to let someone know, such as your GP, but I would tell you if I thought this were the case.

If we use any quotes from you in our reports, you will not be able to be identified.

You can also stop the interview at any time and without giving a reason.

Do you have any questions for me before we start?

**Can you tell me what led to your relative/friend going into hospital and how long they stayed in hospital?**

**Can you tell me about what happened when your relative/friend was in hospital?**

**Whilst they were in hospital, did the healthcare staff discuss with you about stopping one or more of their medicines?**

*Prompt: What do you remember about that conversation?*

**Whilst they were in hospital were any of their medicines stopped?**

*Prompt: How have they been getting on with that?*

**Since your relative/friend left hospital, what has happened?**

**Is there anything we haven’t covered today that you would like to discuss?**

**Would it be OK to call you back in about 3 months' time to talk to you again about any discussions regarding your relatives/friends medication?**

# Consultees 2 [up to 20 minutes]

***Interview 2:***

Introduction:

I am a researcher working on the CHARMER study and we spoke X months ago. The CHARMER trial is testing a method to support doctors and pharmacists to review and stop unnecessary or harmful medicines. I’m interviewing you because you were at one of the hospitals that took part in CHARMER and

I am keen to hear your thoughts on what has happened since your last interview with me – both positive and negative aspects. There are no right or wrong answers. The information you provide will help us to understand what worked well and what could be improved.

Today I will be recording our discussion but only myself, the researchers working on the study and the person who transcribes the interview will hear what we say. When the interview is transcribed, we remove any identifiable information such as your name so that we have an anonymised record of what we talked about. After the interview is transcribed we will securely destroy the recording of the interview.

If you do disclose anything which might identify a risk to yourself or to others, I would have a duty to let someone know, such as your GP, but I would tell you if I thought this were the case.

If we use any quotes from you in our reports, you will not be able to be identified.

You can also stop the interview at any time and without giving a reason.

Do you have any questions for me before we start?

So today I am interested in hearing what has happened since we last met. Last time, your relative/friend had left hospital X weeks before.

**Since we met last for an interview, what has happened?**

**Have you seen your friend’s/family member’s GP or pharmacist since we last spoke?**

**Last time you told me that some of their medicines changed in hospital. Have any of their medicines been re-started since we last spoke?**

**How did you feel about the changes made to their medicines?**

**Part of being in the study has meant that you have phone calls with a nurse, and they ask you about your thoughts and feelings about how your friend/family member’s current health impacts your daily life. How did/do you feel about answering these questions?**

**Is there anything we haven’t covered today that you would like to discuss?**

**Health Innovation East (Post Implementation phase, per step) [up to 60 minutes]**

Introduction: I am a researcher working on the CHARMER study. As you know, the CHARMER study is a programme of research to develop and test an intervention to support geriatricians and pharmacists to proactively deprescribe unnecessary or harmful medicines for older people in hospital.

Today I’d like to ask you about your experience of being involved in the implementation/delivery of the CHARMER study. I am keen to hear your thoughts on how the study went from your perspective – both positive and negative aspects. There are no right or wrong answers. The information you provide will help us to understand what worked well and what could be improved.

Today I will be recording our discussion but only myself, the researchers working on the study and the person who transcribes the interview will hear what we say. When the interview is transcribed, we remove any identifiable information such as your name so that we have an anonymised record of what we talked about. After the interview is transcribed, we will securely destroy the recording of the interview.

If we use any quotes from you in our reports, you will not be able to be identified.

You can also stop the interview at any time and without giving a reason.

Do you have any questions for me before we start?

**Can you tell me a bit about yourself and your role in the CHARMER study?**

**Can you tell me about your experience of being involved in the CHARMER study?**

**How did you find the process of supporting the sites?**

- **Weekly meeting with all sites**
  - **How much time per week was involved in supporting sites?**
  - **How did staff find the resources offered?**
    - **What do you think about the resources offered to staff?**
    - **Any components require more/less support/to what extent**
- **Ad hoc meetings (how often, key issues)**
- **Community of Practice events**
  - **staff sharing experiences with other sites**

**Can you describe any factors that particularly helped or hindered hospital staff in the implementation of the CHARMER intervention?**

**From your perspective, how did the project managers and/or principal investigators respond to CHARMER?**

**Can you describe any factors that particularly helped or hindered you in your role?**

**Is there anything else that you would like to discuss that we have not covered?**
