# Supplementary material for: Implementation of a hospital deprescribing behaviour change intervention, the CompreHensive geriAtRician-led MEdication Review (CHARMER) trial: a process evaluation protocol
Source: BMJ Open. 2026 Jun 2;16(6):e111152. doi: 10.1136/bmjopen-2025-111152 (PMC13239467; doi:10.1136/bmjopen-2025-111152)
Supplement: Supplementary file 5 [file bmjopen-2025-111152.reviewer_comments.pdf]

PEER REVIEW HISTORY

BMJ Open publishes all reviews undertaken for accepted manuscripts. Reviewers are asked to complete a checklist review form and are provided with free text boxes to elaborate on their assessment. These free text comments are reproduced below.

ARTICLE DETAILS

Title (Provisional)

Implementation of a hospital deprescribing behaviour change intervention, the CompreHensive geriAtRician-led MEdication Review (CHARMER) trial: a process evaluation protocol

Authors

Martin-Kerry, Jacqueline M; Bhattacharya, Debi; Taylor, Johanna; Kellar, Ian; Atkins, Bethany; Jones, Charlotte EL; Bywater-Florance, Elizabeth M; Keevil, Victoria; Clark, Allan B; Wright, David; Alldred, David Phillip; Scott, Sion

---

VERSION 1 - REVIEW

**Reviewer**	**1**
**Name**	**Kraut, Roni Y**
**Affiliation**	**Queen's University**
**Date**	**07-Dec-2025**
**COI**	**None**

CHARMER process evaluation protocol review

Dec 7, 2025
It is great that the CHARMER trial has an in-depth process evaluation. Please consider the following points to improve on the description of this evaluation.

Introduction:

- The result of the feasibility study is now in press at Research in Social and Administrative Pharmacy. Please add this reference to the third paragraph in the introduction.
- Please expand on the specific challenges hospitals faced in implementing the intervention as this is directly related to the process evaluation review, ex unfamiliarity with hospital action plan, not being able to implement the deprescribing dashboard, etc.
- The discussion of the theoretical models (6th paragraph) would be better to be fully included in the methods sections, rather than split between the introduction and methods, and this should also help ensure consistency.

- TDF. In the feasibility study protocol (Scott 2023), TDF was used to determine the components of the intervention (prioritized 5 of the 14 domains). The methods section in this manuscript indicates codes will be mapped to TDF to understand the barriers and facilitators. Given it is used to determine the components of the intervention, I'm confused why codes would also be mapped to it?
- NPT. In Scott 2023, NPT is described as being used to identify barriers and facilitators of the intervention. In the methods section in this manuscript, the description is different, it indicates codes will be mapped to NPT to understand how the intervention was received etc. NPT itself has 4 main parts. So please provide more clarification on how NPT will be applied, for instance will mapping be done for each NPT component for each of the process component evaluation in the qualitative analysis?
- Conceptual Framework for Implementation Fidelity is already mentioned in the Fidelity Framework section, so the sentence in the introduction to explain why it was chosen can be moved to that section to keep all the information together.

Methods:

- Line 29 indicates the definitive CHARMER trial began in February 2024. Please indicate when it is expected to be completed and when the process evaluation is expected to start/if it has already started.
- Figure 1 (summary) and Figure 2 (logic model) don't present the components of the intervention in the same order. Please update.
- Process evaluation team. If the coauthors listed on the manuscript are members of the process evaluation team, please indicate this by including their initials.
- Process evaluation review. There are 4 objectives listed in the review but 5 components in Figure 3 – Process Evaluation Component. Please ensure this is consistent. In addition, please adjust the terminology in the text so it is easier to cross-reference between the text and the figure.
- Data sources.
    - I found this somewhat difficult to follow and to link to Figure 3. Consider reorganizing, for instance perhaps include a heading for each component, then put the overall aim for the component and then list the quantitative data sources or perhaps using a table instead. This will also help ensure all data sources are listed on figure 3, for instance I could not find PED-Q on figure 3.
    - Some data sources are provided in the supplementary material but not others (for instance the implementation manual is provided but I couldn't find the project manager questionnaire) and the referencing is inconsistent. If the questionnaire has already been developed, please include it in the supplementary material and reference to their applicable sections.
- Semi-structured interviews.
    - In figure 3, interviews are listed for every component, but the aim in the text does not relate to every component. Please review and reconcile the text with Figure 3.

    - Interview sampling and procedures. Since you mention you would like to examine difference between sites, please clarify whether site will be considered in selecting geriatricians/pharmacists, and patient and carers? This is currently not listed as a criterion.
    - There are several approaches for determining sample size in qualitative research, ex data saturation, informational power. Please indicate what method was used to determine the sample size for patients and carers and primary care practitioners.
    - In the “Hospital and primary care staff participants” section, under “Patient and carer participant”, more detail would be useful on how patients and their carers will be recruited to reduce the chance of selection bias, given only 20-30 are expected to be recruited from many possible patients.
- Quantitative analysis:
    - Analysis is only described for 3 data sources. Please include all the data sources in Figure 3.
    - As you plan to explore differences between sites, you should mention in the analysis that you will be analyzing both in aggregate and subgrouped by site.
    - Will any statistical analysis be done to examine if there is a statistically significant differences between sites, before and after intervention, etc?
- Qualitative analysis
    - Please indicate how trustworthiness (ex. credibility, dependability, confirmability, transferability) will be maintained.
    - This sections states coding will be based on the feasibility study but further down in the “Mapping to theory frameworks” section it states coding will also be based on TDF and NPT. Can these sections be combined and more consistent.
    - This section mentions two researchers will be doing the coding, please clarify whether this will be double-coded and how differences between researchers will be resolved.
- Triangulation. The current description suggests simultaneous triangulation rather than sequential. Please specify which approach is being used and provide a brief rationale for this choice.

VERSION 1 - AUTHOR RESPONSE

Reviewer feedback	Our response
It is great that the CHARMER trial has an in-depth process evaluation. Please consider the following points to improve on the description of this evaluation.	Thank you for this feedback and for your careful review of our manuscript. We have addressed each comment below.
Introduction: The result of the feasibility study is now in press at Research in Social and Administrative Pharmacy.	Thank you, it wasn’t accepted at the time of our submission, but we have now included this reference within the manuscript.

Please add this reference to the third paragraph in the introduction.	
Introduction: Please expand on the specific challenges hospitals faced in implementing the intervention as this is directly related to the process evaluation review, ex unfamiliarity with hospital action plan, not being able to implement the deprescribing dashboard, etc	These findings are published in our feasibility study results paper but we have also included the details on page 3-4, using tracked changes:<br><br>The challenges faced by hospitals when implementing the CHARMER intervention within the feasibility study are reported separately (ref). The main challenges were that sites needed more than the allocated 4 weeks to implement CHARMER, delivering a hospital-level action plan was not achievable, benchmarking reports were not always viewed by geriatricians and pharmacists, delivering the workshops was challenging and some geriatricians did not engage with the workshop videos. We aimed to address these challenges with modification to the implementation period and fund a staff member to lead the implementation at each site, as well as refining guidance about how to implement each of the five CHARMER intervention components.
Introduction: The discussion of the theoretical models (6th paragraph) would be better to be fully included in the methods sections, rather than split between the introduction and methods, and this should also help ensure consistency.	Thank you. We have now moved the theoretical models to the methods, page 13 using tracked changes.
Introduction: TDF. In the feasibility study protocol (Scott 2023), TDF was used to determine the components of the intervention (prioritized 5 of the 14 domains). The methods section in this manuscript indicates codes will be mapped to TDF to understand the barriers and facilitators. Given it is used to determine the components of the intervention, I'm confused why codes would also be mapped to it?	We are evaluating the CHARMER intervention whose components are underpinned by the TDF. Each component is intended to address a barrier or enabler to deprescribing, via a behavioural mechanism i.e. TDF domain. During the evaluation we are exploring whether the intervention components worked via their intended mechanisms or other mechanisms. This is why we are mapping codes to the TDF to understand if the intervention components acted via their intended, or other, mechanisms. We have

	added the following text to the methods page 13) to clarify:<br><br>The CHARMER intervention is underpinned by the Theoretical Domains Framework (TDF), which is a synthesis of behavioural science theories organised into 14 domains e.g., 'Knowledge', 'Beliefs about consequences' and 'Social/professional role and identity'(9). Application of TDF within the CHARMER definitive trial process evaluation permits exploration of the behavioural mechanisms by which the intervention produces its effects and whether this happened via the intended behavioural mechanisms (i.e. TDF domains), and whether any other barriers or enablers are unintentionally addressed by CHARMER. We will explore this by mapping codes describing barriers and enablers to deprescribing to the TDF.
Introduction: NPT. In Scott 2023, NPT is described as being used to identify barriers and facilitators of the intervention. In the methods section in this manuscript, the description is different, it indicates codes will be mapped to NPT to understand how the intervention was received etc. NPT itself has 4 main parts. So please provide more clarification on how NPT will be applied, for instance will mapping be done for each NPT component for each of the process component evaluation in the qualitative analysis?	Thank you for your comment. We are using NPT to understand how the CHARMER intervention was implemented, or not, at each site. We have referenced that we are using the NPT coding framework which has 12 primary constructs, based on the context, mechanism and outcomes realist approach. The mechanisms constructs relate to the original four (coherence, cognitive participation, collective action and reflexive monitoring).<br>We will be mapping the codes to all 12 constructs. Codes used within the feasibility study have already been mapped to NPT but new codes developed for the trial will need to be mapped. We expect that some areas of NPT such as 'sustainment' which comes under 'outcomes' may be more prominent in the trial due to its longer length of duration compared to the 4-week intervention phase in the feasibility study.

Introduction: Conceptual Framework for Implementation Fidelity is already mentioned in the Fidelity Framework section, so the sentence in the introduction to explain why it was chosen can be moved to that section to keep all the information together	We have now moved this to the methods section at the top of page 8.
Methods: Line 29 indicates the definitive CHARMER trial began in February 2024. Please indicate when it is expected to be completed and when the process evaluation is expected to start/if it has already started.	We had included this detail within our cover letter but have now added this to the manuscript on the top of page 5:<br><br>The trial began in February 2024 and will conclude on 31 October 2025. The process evaluation began in February 2024, final data collection will be complete in January 2026, and the analysis will be completed in August 2026.
Methods: Figure 1 (summary) and Figure 2 (logic model) don't present the components of the intervention in the same order. Please update.	We have adjusted the order of the components in the logic model so that they present the components in the same order as Figure 1.
Methods: Process evaluation team. If the coauthors listed on the manuscript are members of the process evaluation team, please indicate this by including their initials.	We have added initials to the process evaluation team paragraph on page 6: The process evaluation team comprises methodologists (JMK, JT, BA, EBF, AC), pharmacists (DB, SS), a geriatrician (VK), and patient and public involvement (PPI) members.
Methods: Process evaluation review. There are 4 objectives listed in the review but 5 components in Figure 3 – Process Evaluation Component. Please ensure this is 2 consistent. In addition, please adjust the terminology in the text so it is easier to cross-reference between the text and the figure.	Thank you for this comment. Figure 3 shows an overview of the aspects we will explore in the process evaluation. We have updated the objectives on page 6, process evaluation overview to include a fifth objective, namely 'explore the perceived effect of the intervention'.
Methods:<br>Data sources. I found this somewhat difficult to follow and to link to Figure 3. Consider reorganizing, for instance perhaps include a heading for each component, then put the overall aim for the component and then list the quantitative data sources or perhaps using a table instead. This will also help ensure all data sources are listed on	 We have used this figure previously (Scott et al. CompreHensive geriAtRician-led MEdication Review (CHARMER): protocol for a feasibility study of a hospital deprescribing behaviour change intervention. BMJ Open 2023;**13:**e075795. doi: 10.1136/bmjopen-2023-075795)

figure 3, for instance I could not find PED-Q on figure 3.	We would like to include the figure as it is, as it summarises the different data sources by quantitative and qualitative and how they will help us understand each component listed at the top of the figure.<br>We have cross-checked to ensure all data sources are included in the figure – only PED-Q was missing but is now included.
Methods:<br>Data sources. Some data sources are provided in the supplementary material but not others (for instance the implementation manual is provided but I couldn't find the project manager questionnaire) and the referencing is inconsistent. If the questionnaire has already been developed, please include it in the supplementary material and reference to their applicable sections	We have now also included the following within the supplementary files:<br>• Project Manager questionnaire<br>• Site profile questionnaire<br>• Principal Investigator bi-monthly questionnaire<br>• PED-Q
Methods:<br>Semi-structured interviews. In figure 3, interviews are listed for every component, but the aim in the text does not relate to every component. Please review and reconcile the text with Figure 3	We have added additional text to the aims on page 10 to clarify this and ensure it correlates with Figure 3 which provides more high-level information about the interviews under each component.<br><br>**Aims:**<br>To understand implementation, how the intervention was received and any perceived impact of the intervention and any contextual factors that may influence this (PIs, project managers and participating geriatricians and pharmacists -all intervention sites)<br><br>To understand geriatrician and pharmacist recruitment and participation (PI, project managers, and participating geriatricians and pharmacists -all intervention sites)<br>To understand patient/carer recruitment and data collection processes (research delivery teams - all sites) To explore patients' and their carers' experience of having a medicine(s) stopped in hospital

	(patients and carers- all intervention sites)<br>To understand the impact of CHARMER and hospital deprescribing in general on primary care (primary care)<br><br><br>We have decided not to add which interviews relate to each component in Figure 3 as this would make the figure very text-heavy and we hope that the revised aims in the text under semi-structured interviews clarifies this.
Methods:<br>Semi-structured interviews.<br>Interview sampling and procedures.<br>Since you mention you would like to examine difference between sites, please clarify whether site will be considered in selecting geriatricians/pharmacists, and patient and carers? This is currently not listed as a criterion	 We are undertaking interviews at all sites to understand what happened at each site. This will be a large, complex piece of work but we decided to do this approach rather than a priori sample sites. We have not made any changes to the text as the sub-heading 'semi-structured interviews' states that interviews will happen at all sites.
Methods: Semi-structured interviews.<br>There are several approaches for determining sample size in qualitative research, ex data saturation, informational power. Please indicate what method was used to determine the sample size for patients and carers and primary care practitioners	We will only be interviewing patients and carers in the enhanced data collection period after the intervention has been received by participating geriatricians and pharmacists.<br>We note that there are different approaches to sampling and we wanted to ensure that we heard from a wide range of patients and carers. We decided to aim for 20-30 patients/carers across the trial, noting that not all patients and carers would consent to the optional interview and not all patients would have a medicine(s) stopped in the trial. We planned to continue interviewing if new data was still arising in interviews.<br><br>For primary care interviews, we aimed to interview 10-15 practitioners and again this was determined based on recruitment of primary care practitioners needing to have at least one patient participate in CHARMER and have a medicine(s) stopped whilst in the trial. We

	are focusing on achieving a wide range of primary care perspectives through our sampling (e.g. location, demographics of primary care practitioner).
Methods: Semi-structured interviews.<br>In the “Hospital and primary care staff participants” section, under “Patient and carer participant”, more detail would be useful on how patients and their carers will be recruited to reduce the chance of selection bias, given only 20-30 are expected to be recruited from many possible patients.	Research delivery teams are approaching patients and carers to participate in the trial. When consenting, patients and carers can choose to be involved in an interview (optional activity). From those who consent to being approached by the process evaluation team to undertake an interview, we will contact patients and carers to participate in an interview, aiming to achieve variation in demographics (such as age, gender, ethnicity, location, medicines stopped).<br>We know from the feasibility study that not all patients who consent to be approached about an interview, will be able to undertake the interview after discharge (e.g. readmission to hospital soon after discharge, difficulty reaching by telephone) but we will follow up and seek to obtain perspectives from a wide range of patients and carers.<br>We will also have the PED-Q responses for a larger number of patients and carers to ensure we have a good understanding of the patient and carer perspectives of having a medicine(s) stopped in hospital.
Methods:<br>Quantitative analysis<br>Analysis is only described for 3 data sources. Please include all the data sources in Figure 3.	We have added in analysis of the PED-Q and the site profile questionnaire and bi-monthly PI questionnaires within the methods section, using tracked changes.
Methods:<br>Quantitative analysis<br>As you plan to explore differences between sites, you should mention in the analysis that you will be analyzing both in aggregate and subgrouped by site.	It is unclear what the reviewer means by ‘aggregate and subgrouped by site’.<br>We plan to do descriptive by site analysis for the fidelity data which is collected at the site level. Any outcome which is collected at an individual level will be summarised by site and these compared across sites.<br><br>We do not plan to do any analysis subgrouped by site.

Methods:<br>Quantitative analysis<br>Will any statistical analysis be done to examine if there is a statistically significant differences between sites, before and after intervention, etc?	We have added text to the paper on page 12 to address the analysis of the process of de-prescribing:<br><br>The analysis conducted will be a Mann Whitney test of the change in the response between pre and post intervention.<br><br>All the other analyses will be descriptive for the process evaluation.
Methods:<br>Qualitative analysis<br>Please indicate how trustworthiness (ex. credibility, dependability, confirmability, transferability) will be maintained.	In the methods we have added more detail to state:<br>Weekly process evaluation meetings throughout the data collection and analysis periods including intervention, clinical and methodological expertise, and regular input from PPI members, will be undertaken. These meetings will be used to discuss the data and review supporting quotes-underlying codes, and to address queries that arise during the analysis, to increase rigour and consistency within the analysis.
Methods:<br>Qualitative analysis<br>This sections states coding will be based on the feasibility study but further down in the "Mapping to theory frameworks" section it states coding will also be based on TDF and NPT. Can these sections be combined and more consistent	The codes will be based on codes refined from the feasibility study. We will subsequently map these codes to both TDF and NPT after the coding is complete. That is, the transcripts are coded (using the refined feasibility study codes) then these codes will be mapped to TDF and NPT to provide further understanding of what happened.
Methods:<br>Qualitative analysis<br>This section mentions two researchers will be doing the coding, please clarify whether this will be double-coded and how differences between researchers will be resolved	We will not be double coding. We will refine the coding framework to ensure we have a consistent set of codes and definitions. We will meet regularly to discuss coding and supporting quotes for different codes to check we are coding similarly.
Methods:<br>Triangulation.<br>The current description suggests simultaneous triangulation rather than	We plan to triangulate data as we analyse different data sources and want to explore these findings further. For example, if we

sequential. Please specify which approach is being used and provide a brief rationale for this choice	identify in interviews different perspectives about the intervention, we would like to triangulate these to give a better understanding of the process from all perspectives. Where we find interesting findings from quantitative data, we will seek to triangulate with relevant qualitative data to explore the reasons for this.<br><br>We have added to the manuscript on page 14 to clarify this:<br>The triangulation process will be decided iteratively based on what we find when analysing separate data sources to enable us to explore this in more detail.

VERSION 2 - REVIEW

**Reviewer**	**1**
**Name**	**Kraut, Roni Y**
**Affiliation**	**Queen's University**
**Date**	**15-Mar-2026**
**COI**	

Thank you for the updates you made to your manuscript.

Please review the following items:

1. Abstract. The abstracts mentions “quantitative data will be analysed descriptively”, however the manuscript has been updated to include the Mann Whitney test, so this sentence in the abstract should be updated.

2. Methods. Data source section. The point was not to update figure 3 but rather to update the organization of this section to be more consistent with figure 3. Unless there is a reason for the current order of the data sources in the manuscript, it would be better to list them in the same order as the figure, with the same terminology, so it is easier to follow. For instance, the first four data sources (besides interview) in Figure 3 are “recording of implementation”, “implementation manual”, “fidelity framework” and “site profile questionnaire” but in the text the order of the data sources is implementation manual”, “recording of CHARMER implementation”, “Project manager questionnaire” and “Fidelity framework”.

3. Methods: Semi-structured interviews. Your response indicates you will be using the data saturation approach to determine the sample size for your interviews, However, this was not updated in the manuscript.

4. Methods: Qualitative analysis. Trustworthiness was not fully addressed with the added text. Please indicate specifically how credibility, dependability, confirmability, reflexivity, and transferability will be addressed.

VERSION 2 - AUTHOR RESPONSE

Reviewer feedback	Our response
1. Abstract. The abstracts mentions "quantitative data will be analysed descriptively", however the manuscript has been updated to include the Mann Whitney test, so this sentence in the abstract should be updated.	Thank for noticing this. We have addressed this in the abstract with:<br><br>Most quantitative data will be analysed descriptively; however, changes in staff questionnaire responses pre- and post-intervention will be analysed using a Mann-Whitney test.
2. Methods. Data source section. The point was not to update figure 3 but rather to update the organization of this section to be more consistent with figure 3. Unless there is a reason for the current order of the data sources in the manuscript, it would be better to list them in the same order as the figure, with the same terminology, so it is easier to follow. For instance, the first four data sources (besides interview) in Figure 3 are "recording of implementation", "implementation manual", "fidelity framework" and "site profile questionnaire" but in the text the order of the data sources is implementation manual", "recording of CHARMER implementation", "Project manager questionnaire" and "Fidelity framework".	Thank you for this feedback. We have addressed this by ensuring the wording and flow within the figure exactly matches the wording in the text. Using tracked changes, we have moved the text to match the order the data sources appear within the figure. The terminology in the figure and the manuscript text now matches.<br>During this process we have decided to have 'contextual factors' as an aspect that goes across the top of the figure as context will influence all of the other components within the figure. We feel that the messaging is clearer for this change. It also addresses your comments about the order of information in the figure and the text.
3. Methods: Semi-structured interviews. Your response indicates you will be using the data saturation approach to determine the sample size for your interviews, However, this was not updated in the manuscript.	We have added the following sentence to the section labelled 'Interview sampling and procedures':<br>Sampling will be undertaken to ensure we hear from a wide range of patients, carers and staff. We will continue interviews until data saturation is reached which we define as no new data relating to

	answering our research objectives are arising from interviews.
4. Methods: Qualitative analysis. Trustworthiness was not fully addressed with the added text. Please indicate specifically how credibility, dependability, confirmability, reflexivity, and transferability will be addressed.	We have added the following text to the qualitative analysis section:<br>To ensure trustworthiness of the data analysis, we will incorporate a number of approaches. Weekly process evaluation meetings throughout the data collection and analysis periods including intervention, clinical and methodological expertise, and regular input from PPI members, will be undertaken. These meetings will be used to discuss the data and review supporting quotes underlying codes, and to address queries that arise during the analysis, to increase rigour and consistency within the analysis. This process will ensure the analysis is credible. Both coders (JMK, EBF) will also maintain reflexive diaries about their thoughts on the data and any biases or focus they are aware of when coding to ensure confirmability. Their background and how this has influenced their data collection and analysis will be recorded to capture and report reflexivity and the role this has played in the analysis process. We will maintain an audit trail of all research steps, decisions and methodological changes made during the analysis to ensure dependability of the analysis. Finally, we will ensure we report data across all sites, including contextual aspects, to enable transferability of the findings.
